# Transcriptional analysis of cell growth and morphogenesis in the unicellular green alga *Micrasterias *(Streptophyta), with emphasis on the role of expansin

**DOI:** 10.1186/1471-2229-11-128

**Published:** 2011-09-25

**Authors:** Katrijn Vannerum, Marie JJ Huysman, Riet De Rycke, Marnik Vuylsteke, Frederik Leliaert, Jacob Pollier, Ursula Lütz-Meindl, Jeroen Gillard, Lieven De Veylder, Alain Goossens, Dirk Inzé, Wim Vyverman

**Affiliations:** 1 Laboratory of Protistology and Aquatic Ecology, Department of Biology, Ghent University, 9000 Gent, Belgium; 2 Department of Plant Systems Biology, VIB, 9052 Gent, Belgium; 3 Department of Plant Biotechnology and Bioinformatics, Ghent University, 9052 Gent, Belgium; 4 Phycology Research Group, Department of Biology, Ghent University, 9000 Gent, Belgium; 5 Plant Physiology Division, Cell Biology Department, University of Salzburg, 5020 Salzburg, Austria

## Abstract

**Background:**

Streptophyte green algae share several characteristics of cell growth and cell wall formation with their relatives, the embryophytic land plants. The multilobed cell wall of *Micrasterias denticulata *that rebuilds symmetrically after cell division and consists of pectin and cellulose, makes this unicellular streptophyte alga an interesting model system to study the molecular controls on cell shape and cell wall formation in green plants.

**Results:**

Genome-wide transcript expression profiling of synchronously growing cells identified 107 genes of which the expression correlated with the growth phase. Four transcripts showed high similarity to expansins that had not been examined previously in green algae. Phylogenetic analysis suggests that these genes are most closely related to the plant EXPANSIN A family, although their domain organization is very divergent. A GFP-tagged version of the expansin-resembling protein MdEXP2 localized to the cell wall and in Golgi-derived vesicles. Overexpression phenotypes ranged from lobe elongation to loss of growth polarity and planarity. These results indicate that MdEXP2 can alter the cell wall structure and, thus, might have a function related to that of land plant expansins during cell morphogenesis.

**Conclusions:**

Our study demonstrates the potential of *M. denticulata *as a unicellular model system, in which cell growth mechanisms have been discovered similar to those in land plants. Additionally, evidence is provided that the evolutionary origins of many cell wall components and regulatory genes in embryophytes precede the colonization of land.

## Background

Although the form and function of plant cells are strongly correlated, the processes that determine the cell shape remain largely unknown. Plant cell morphogenesis is regulated in a non-cell-autonomous fashion by the surrounding tissues [1], hormone interference during ontogenesis, and sometimes by polyploidy as a consequence of endoreduplication [2,3]. In contrast, in unicellular relatives of land plants, it is possible to study the endogenous controls of cell morphogenesis without the interference by interacting cells and to better understand how these mechanisms have evolved in the green lineage.

**Figure 1:**
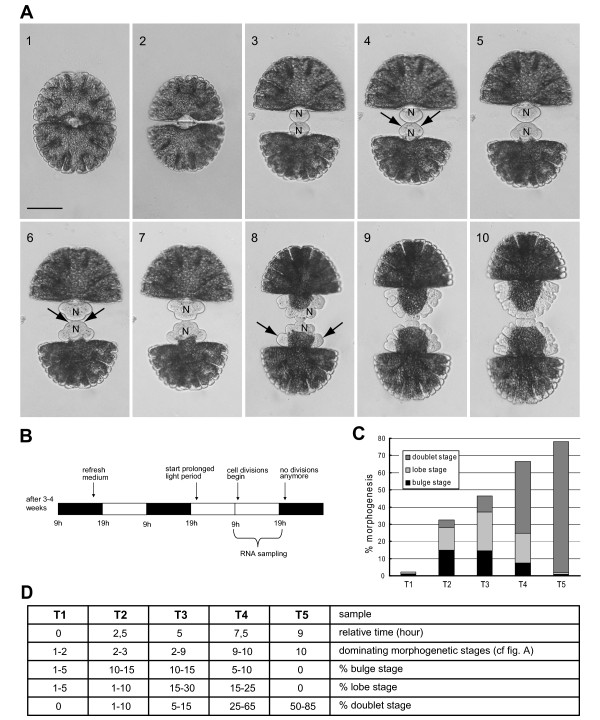
**Morphogenesis of *Micrasterias denticulata *and distribution of morphogenetic stages in the synchronized sample series**. (A) Morphogenesis of *M. denticulata*. (1) Vegetative cell. (2) During mitosis, a septum originating from the cell wall girdle grows inward centripetally, taking 15-20 min. (3) Bulge stage; the septum bulges uniformly. (4) Development of the first pair of indentations (arrows), ~75 min after septum completion. (5) Three-lobed stage. (6) Development of the second pair of indentations (arrows). (7) Five-lobed stage. (8) Doubling of the lateral lobes (arrows). (9) Formation of further indentations and lobe tips, followed by the doublet stage. N, Nucleus. Note the migration of the nucleus during cell growth. Scale bar = 100 μm. (B) Scheme of the synchronization protocol. After 3-4 weeks, a stationary culture is obtained and the growth medium is refreshed, concomitantly with the reduction in cell density, shortly before the beginning of the light period of that day. The majority of the cells divide in the second dark period afterward. This dark period is replaced by a light period and sampled. Black, dark period; white, light period. (C), Distribution of morphogenetic stages in the RNA samples for cDNA-AFLP, replication 1. (D) Table representing the characteristics of the samples used for cDNA-AFLP (replications 1 and 2) and real-time qPCR.

The desmid *Micrasterias denticulata *is a member of the conjugating green algae (Zygnematophyceae) that comprise the closest extant unicellular relatives of land plants [4-8]. *M. denticulata *cells consist of two bilaterally symmetrical flat semicells, notched deeply around their perimeter into one polar lobe and four main lateral lobes. Following cell division, each semicell builds a new one through a process of septum bulging and symmetrical local growth cessations to form the successive lobes (Figure 1A). After completion of the primary wall (during the doublet stage), a rigid cellulosic secondary cell wall pierced by pores is deposited, followed by shedding of it. This peculiar growth mechanism makes *Micrasterias *an ideal model to study the spatial and temporal patterning of cell wall biogenesis [9].

Ultimately, the plant cell morphology is determined by the composition and structure of the cell wall that governs the cell expansion direction and rate. As in land plants, the primary cell wall of *M. denticulata *Bréb. consists mainly of pectins [10,11], cellulose microfibrils [12], hemicelluloses [13] and arabinogalactan proteins (AGPs) [10,13]. The secondary cell wall owes it rigidness to cellulose microfibrils originating from rosettes organized as hexagonal arrays [14,15], whereas mixed-linked glucan is the dominant hemicellulose [13].

In land plants, expansins are important regulators of turgor-driven cell wall expansion. These cell wall proteins comprise a large multigene superfamily consisting of four families (EXPA, EXPB, EXLA and EXLB) of which the evolutionary relationships are well characterized [16,17]. They are unique in their ability to loosen the cell wall non-enzymatically by disrupting hydrogen bonds that link the cellulose and hemicellulose wall components [18-21]. Land plant expansins consist of two domains and a secretion signal. The N-terminal expansin domain 1 and the C-terminal expansin domain 2 are homologous to the catalytic domain of glycoside hydrolase family 45 (GH45) proteins and a domain present in a family of grass pollen allergens, identified as a putative cellulose binding site [22], respectively. Expansins play a role in tissue development [23,24] and in growth of suspension-cultured cells [25,26]. Although genes encoding expansin-like proteins have been recently identified in green algae transcriptomes [27], their physiological function and phylogenetic relationships with land plant expansins remain unknown.

Here, we explore the molecular basis of cell morphogenesis and cell wall formation in synchronized *M. denticulata *cells by means of a cDNA-amplified fragment length polymorphism (cDNA-AFLP)-based quantitative transcriptome analysis [28]. Several cell wall-related genes, among which expansins, were identified. Examination of the expansins provided the first structural, phylogenetic and functional data on green algal homologues within this gene family.

## Results

### cDNA-AFLP expression profiling

**Figure 2:**
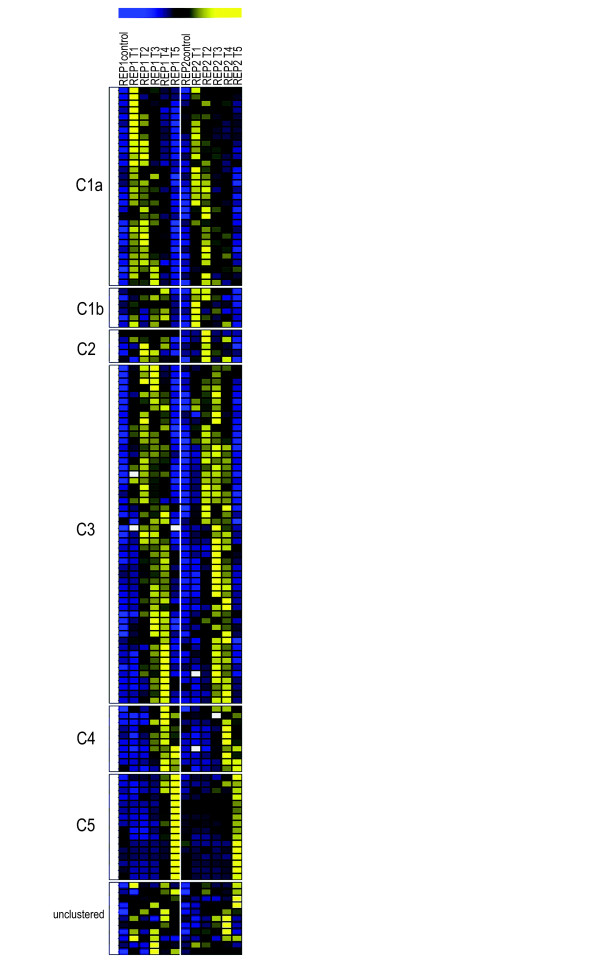
**Adaptive quality-based clustering of annotated cell growth-modulated TDFs**. Each row represents the relative transcript accumulation measured for each TDF across the two replicated time series. Yellow and blue, transcriptional activation and repression relative to the average expression level over the time course, respectively; white, missing data. Cluster names (C1 to C5) are indicated on the left.

**Figure 3:**
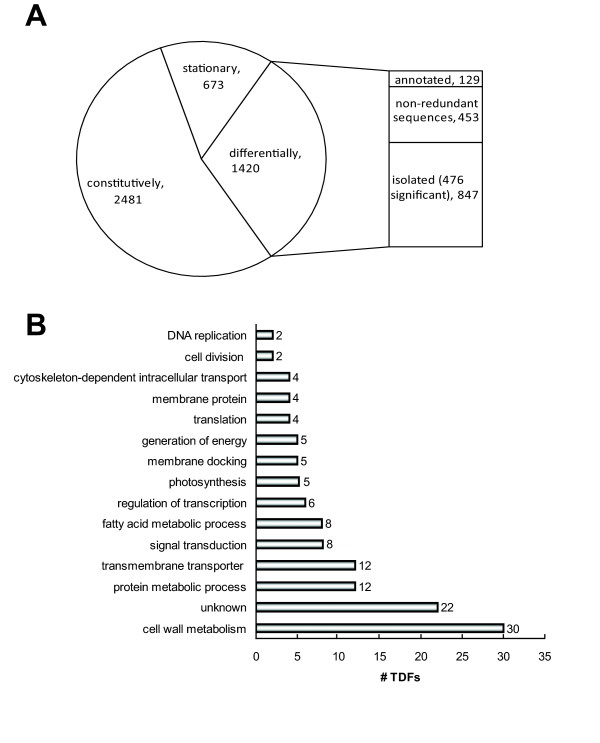
**Transcript derived fragments (TDFs) identified by cDNA-AFLP analysis of *Micrasterias denticulata *cell growth**. (A) In total, 4574 TDFs were scored, of which 2481 were constitutively expressed, 673 only in stationary cultures and 1420 displayed altered expression patterns across time (476 significantly; *P *< 0.009; *Q *< 0.05). Of the latter group, 847 were isolated from gel. From 453 non-redundant sequences, 129 could be annotated. (B) Functional classification of the 129 annotated transcript-derived fragments (TDFs) differentially modulated during cell growth.

First we developed a synchronization protocol to monitor the cell morphogenesis-related gene expression in *M. denticulata*. The protocol was based on the observation that the majority of the cells grown in a 14-h light/10-h dark regime divided during the second dark period, after the growth medium of a stationary culture (obtained after 3-4 weeks) had been refreshed and, concomitantly, the cell density reduced at the start of the light period. Replacing the dark period by a light period enhanced the amount of synchronically dividing cells (Figure 1B). The effect of cell density on synchronization was significant (GLM; *F*-test; *P *< 0.001), with an optimal cell density below 80 cells mL^-1^. Following synchronization, up to 85% of the cell population divided during an 8- to 9-h period, showing a sigmoid course (Figure 1C,D; Additional file 1). By sampling this period at five consecutive time points we obtained samples with different proportions of cells at the major morphogenetic stages (Figure 1A,C,D). cDNA-AFLP expression profiling of these samples allowed the assignment of differentially expressed genes to either the onset of cell division (Figure 1A2; Figure 2 (C1a and C1b)), the bulge (Figure 1A3; Figure 2 (C2)), the lobe (Figure. 1A4-A9; Figure 2 (C3)), or the doublet stage, during which the secondary cell wall is formed (Figure 2 (C4 and C5)). In total, the relative abundance was monitored of 4574 transcript-derived fragments (TDFs) during the cell growth of *M. denticulata *(Figure 3, Additional file 2), for which the expression patterns were altered visibly across time in 1420 and significantly (*P*<0.009; *Q*<0.05) in 476 TDFs. According to other studies [29,30], we estimate that two-thirds of the mRNA population was sampled, implying that the real number of genes differentially expressed during cell growth of *M. denticulata *could be ~2100. A high similarity (E-value < 1.E-01 and similarity >50%) to database entries with assigned identities and unknown or hypothetical genes was found for 107 and 22 TDFs, respectively, mostly with Embryophytes and not with Chlorophyta. However, the majority of the TDFs (324 or 71.5%) showed no sequence similarity to any database entry (Figure 3; Additional file 3). Plausible explanations might be sequences too short to reveal any significant identity, short sequences representing non-conserved portions of genes, TDFs originating from the 3'-untranslated region of a gene, or TDFs representing genes specific to *M. denticulata *or streptophytic algae.

Of the 129 annotated genes, 118 clustered into six groups (designated C1a, C1b, C2, C3, C4, and C5) (Figure 2) according to the timing of their highest expression (Figure 1C,D). Except for one cluster consisting of six genes (cluster C1b; Figure 2), the expression profiles were reproducible in the two independent sampling series. The few genes not included in one of the described clusters typically showed narrow temporal expression patterns.

Based on their annotation, the TDFs were classified into 14 functional categories, named according to the Gene Ontology terminology (http://www.geneontology.org) (Figure 3; Additional file 3). The association between the functional category and the TDF clustering was not significant (χ^2 ^test; *p *= 0.070). The major group with a significant hit was involved in cell wall metabolism. The second largest category corresponded to sequences sharing significant similarity to unknown or hypothetical proteins.

Of 18 TDFs with similarity to genes involved in cell wall biogenesis or cell pattern formation, the RNA samples of the second cDNA-AFLP replication series and on an independently sampled series (Additional file 1) were analyzed by real-time quantitative reverse-transcription (qRT)-PCR. In general, the expression profiles obtained by cDNA-AFLP and qRT-PCR (Additional file 4) corresponded well (Additional file 5), confirming the obtained expression results.

### Genes relevant for cell pattern formation

Seven TDFs could be identified that might be relevant for cell pattern formation in *M. denticulata*, among which two members of the Rab GTPase cycle and two members of the SNARE cycle of membrane fusion reactions. Rab8, similar to *Md*1852, is known to be involved in post-Golgi transport to the plasma membrane, inducing the formation of new surface extensions and believed to be regulated by a guanine nucleotide dissociation inhibitor [31] possibly corresponding to *Md*0818. Both *Md*1852 and *Md*0818 belonged to cluster C1a and, thus, had increased mRNA levels before the onset of mitosis. This observation might be related to the determination of the basic symmetry of a *M. denticulata *cell before mitosis, indicated by the development of a three-lobed semicell after removal of the nucleus [32]. In contrast, the SNARE cycle members were highly expressed in cluster C3, pointing to a role in further differentiation during the lobe stages for *Md*1404 (similar to plant syntaxin 32) and *Md*1560 (similar to a regulatory AAA-type of ATPase).

Two TDFs were identified encoding putative glycophosphatidylinositol (GPI) anchors: *Md*4071 and *Md*4341, belonging to clusters C1a, and C4, respectively. Among other properties, the function of a GPI anchor might be its dominant targeting to a specific membrane domain [33], possibly establishing a membrane template for morphogenesis. *Md*4341 turned out to be a 179-amino-acid protein containing a signal peptide and a fasciclin domain (a putative cell adhesion domain) (E-value 2.9E-07), with similarity to a fasciclin-like and an AGP-like protein from *Brachypodium sylvaticum *[CAJ26371.1] and *Arabidopsis thaliana *[AAM62616.1], respectively (Additional file 6).

*Md*3533 (cluster C3), similar to a very-long-chain fatty acid-condensing enzyme, might be involved in morphogenesis in accordance to the essential role in cell expansion during plant morphogenesis of *Arabidopsis *[34].

### Genes involved in cell wall metabolism

**Figure 4:**
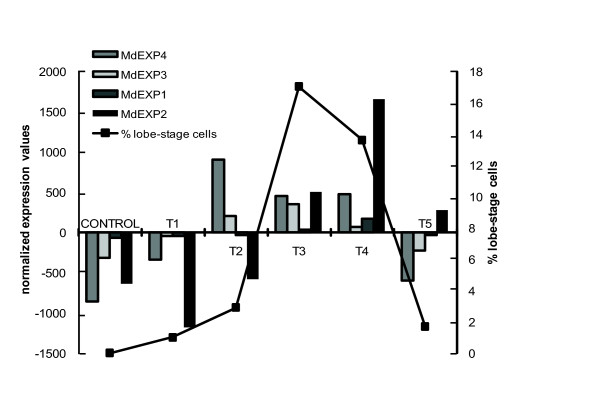
**Normalized cDNA-AFLP expression values of *Micrasterias denticulata *expansin-resembling proteins in synchronized cultures in relation to the proportion of lobe-forming cells in these cultures**. The samples (T1-T5) are defined in Figure 1D.

A total of 30 cell wall-related genes were identified. Six TDFs operating in the monosaccharide metabolism, evenly distributed over C1 and C3, could be identified as UDP-pyrophosphorylases (*Md*1739, *Md*2333, and *Md*2565), a phosphoglucomutase (*Md*2842), a rhamnose synthase (*Md*1089), and a GDP-mannose 3,5-epimerase (*Md*3053). Nine polysaccharide synthesis enzymes all nearly clustered in C3, among which two cellulose synthases, *Md*0757 (see also [35]) and *Md*3668, and one cellulose synthase-like (*CSL*) gene of the *CSLC *family, *Md*2838. The exostosin family glycosyltransferases *Md*0450, *Md*1114, *Md*2144, and the glycosyltransferase *Md*0257 might synthesize the hemicellulosic or pectinous part of the cell wall and mucilage as well that is pectic in nature [11] and secreted simultaneously with cell wall material during cell growth [36]. *Md*3598 was the α-1,6-xylosyltransferase, typical of the hemicellulose biosynthetic pathway, whereas *Md*0888 was the xyloglucan endotransglycosylase/hydrolase (XET/XTH) that is a xyloglucan-modifying enzyme. The open reading frame (ORF) of *Md*0888 encoded a 277-amino-acid protein with a signal peptide and a GH16-XET domain (E-value 6.10E-37) and therefore designated MdXTH1. The catalytic site DEIDFEFLG, conserved among GH16 family members [37] and most seed plant XTHs [38] was present in MdXTH1 as xExDxEFxG and immediately followed by a potential N-glycosylation site NxT/S [39] (Additional file 7). The other 15 identified TDFs were involved in wall assembly, reorganization, and selective degradation. Four of them gave significant hits with expansins: *MdEXP1 *(C4), *MdEXP2 *(C4), *MdEXP3 *(C3), and *MdEXP4 *(C3). Whereas *MdEXP4 *and *MdEXP3 *were expressed during the early morphogenetic stages (C3), *MdEXP1 *and *MdEXP2 *were up-regulated during later stages (C4) (Figure 4). Changes in the internal structure of the cell walls, required for cell expansion, might be achieved by the release of hydroxyl radicals mediated by the class-III peroxidases *Md*0434 and *Md*0493. Peroxidase-generated hydroxyl radicals could cause non-enzymatic wall loosening by cleavage of various polysaccharides [40]. The ORF of *Md*0434 contained a secretion signal peptide and a Pfam peroxidase domain (E-value 2.50E-97) (Additional file 8). The H_2_O_2 _substrate for the peroxidase activity was probably generated by the glyoxal oxidases *Md*0606, *Md*1709, and *Md*3495. Hydrolytic enzymes included the pectinesterase *Md*4415, the endo-β-1,6-galactanase *Md*1480, and two members of cluster C5: the polygalacturonidase *Md*3500 and the β-glucosidase *Md*0559, possibly involved in degradation of a connecting zone between the primary and the secondary cell wall, thereby enabling shedding of the primary cell wall [41].

### Phylogenetic relationship of *M. denticulata *expansin-resembling proteins

**Figure 5:**
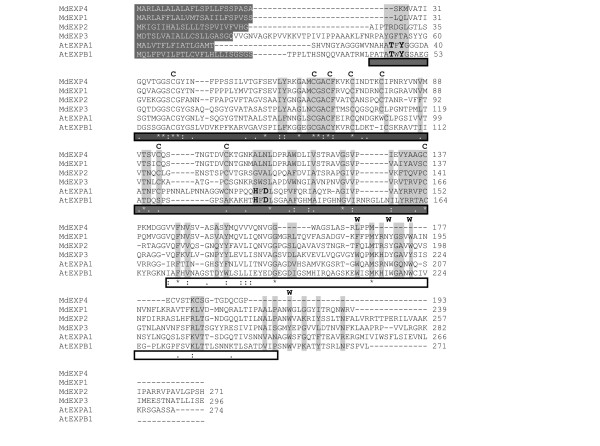
**Alignment of the amino acid sequence of the *Micrasterias denticulata *expansin-resembling proteins **Alignment of the amino acid sequence of the *M. denticulata *expansin-resembling proteins MdEXP2, MdEXP1, MdEXP4, and MdEXP3 with the *Arabidopsis thaliana *EXPA1 [NP_001117573] and EXPB1 [NP_179668]. The C-terminal extension of MdEXP2 is omitted (see Additional file 11). Dark-shaded white characters represent N-terminal sorting signals. Dark gray and white boxes below the alignment indicate the expansin domains 1 and 2, respectively. Conserved Cys (C) and Trp (W) residues are indicated above the alignment. The key residues of the GH45 catalytic site that are conserved in domain 1 of the EXPA and EXPB expansin families are shown in bold. Conserved expansin residues and motifs are lightly shaded. Asterisks mark identical residues; colons and periods indicate full conservation of strong and weak groups, respectively.

As the involvement of expansins in cell growth of green algae had not been examined previously, we concentrated the experiments on this class of proteins. The full length characteristics of the *M. denticulata *expansin-resembling proteins (*Md*EXPs) are given in Additional file 9. *MdEXP1 *and *MdEXP4 *exhibited the highest sequence similarity (74% identity, 84% similarity) (Figure 5).

**Figure 6:**
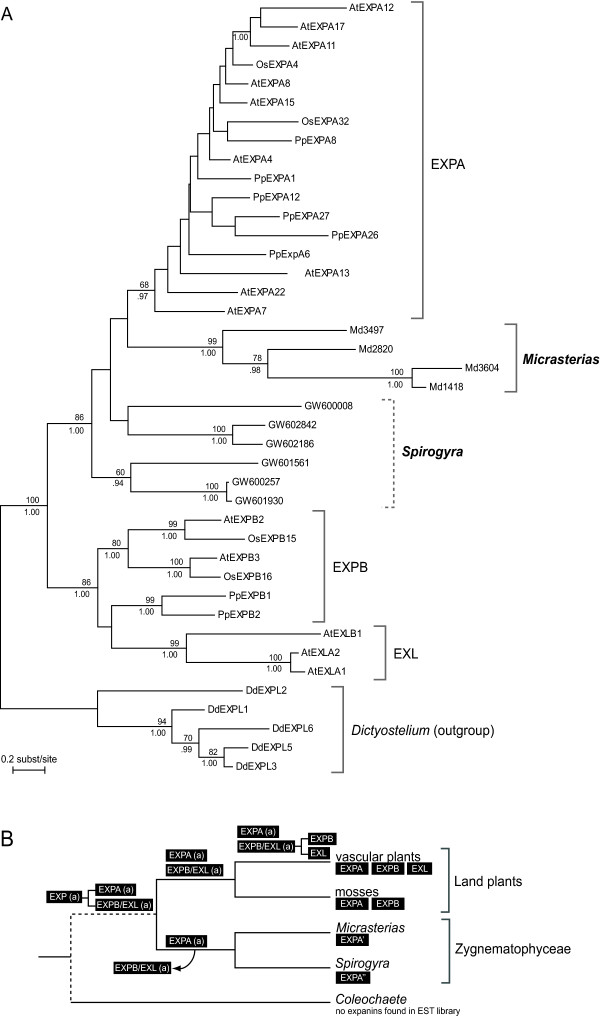
**Maximum likelihood phylogeny of the plant expansin gene family **(A) Maximum likelihood (ML) phylogeny of the plant expansin gene family, showing the phylogenetic position of the *Micrasterias *and *Spirogyra *genes. Numbers at nodes indicate ML bootstrap values (top) and Bayesian posterior probabilities (bottom); values below 50 and 0.9, respectively, are not shown. Dd, *Dictyostelium discoideum *(outgroup); Pp, *Physcomitrella patens*; Os, *Oryza sativa*; At, *Arabidopsis thaliana*. (B) Possible events hypothetically explaining the distribution of expansin gene families in land plants and Zygnematophyceae. The organismal tree is based on multigene phylogenetic analyses [5,6] and only includes taxa in which expansins have been found, along with *Coleochaete *that apparently lacks expansins based on transcriptome analyses [27]. The dotted line in the tree indicates phylogenetic uncertainty. "(a)" marks ancestral gene families, EXPA' and EXPA'' represent the EXPA-related genes found in *Micrasterias *and *Spirogyra *respectively.

Phylogenetic analysis of the first dataset revealed that all *Md*EXPs were recovered as a monophyletic group with high support (BV = 99, PP = 1.00) (Figure 6A). The *Micrasterias *and *Spirogyra *sequences fell within the plant expansins and were most closely related to the EXPA family, with which they formed a well supported clade (BV = 86, PP = 1.00). The *Md*EXPs are recovered sister to the EXPA clade and the *Spirogyra *sequences form a paraphyletic assemblage, but the relationships between the *Micrasterias *and *Spirogyra *expansins and the EXPA clade are poorly supported. The high sequence divergence of expansins within and among *Micrasterias *and *Spirogyra *is shown by the relatively longer branches than those within the EXPA clade. In the second dataset, the putative expansin sequences of Chlorophyta formed a highly divergent clade, separated from the plant expansins by a very long branch (Additional file 10). Although the relationships between the Chlorophyta clade, the *Dictyostelium *clade and the plant expansin families were poorly resolved, the phylogenetic position of the *Micrasterias *clade, closely allied to the EXPA family, was well supported.

### Domain organization of the *M. denticulata *expansin-resembling proteins

**Figure 7:**
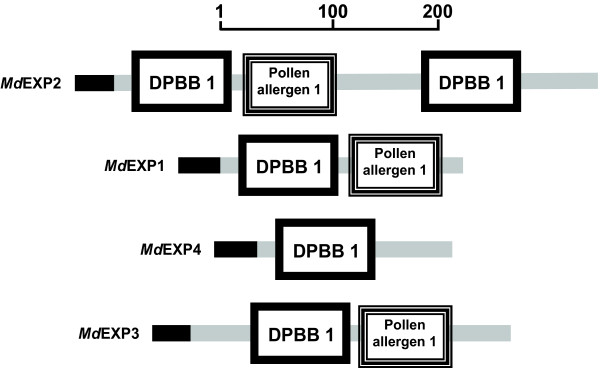
**Schematic representation of the domains with significant E-value in the *Micrasterias denticulata *expansin-resembling proteins MdEXP2, MdEXP1, MdEXP4, and MdEXP3**. The black line indicates the signal peptide. DPBB1, a rare lipoprotein A-like double-psi beta-barrel domain. The pollen allergen 1 domain is similar to expansin domain 2. Scale gives length in amino acids.

The structural domain organization of the different *Md*EXPs was compared with the characteristic structural features of plant expansins (Table 1, Figure 5, Figure 7). A secretion signal peptide was present in all of them (Figure 5, Figure 7, Table 1). While the pollen-allerg-1 domain occurred in all proteins, except MdEXP4, the GH45 domain was found in MdEXP2 and MdEXP3 only, albeit with insignificant E-values. Nevertheless, in all sequences, a DPBB-1 domain was present, a rare lipoprotein A-like double-psi beta-barrel, to which GH45 belongs, and even twice in *MdEXP2 *(Additional file 11). The eight cystenyl residues forming disulfide bridges in fungal GH45 enzymes and maintaining their folded structure [16] were conserved in the expansin domain 1 of some of the plant expansin groups [22] and also in the *Md*EXPs (Figure 5). In *M. denticulata*, the GGACGY motif was present as GGSCGY/F, whereas the GxxCGxCF/Y motif in the same expansin domain 1 was fully conserved. A third motif characteristic for this domain, the Y/FRRVPC motif, varied among the *Md*EXPs (Table 1). The key residues of the GH45 catalytic site, conserved among EXPA and EXPB proteins (see Figure 5, indicated in bold), were absent. In land plant expansins, the pollen-allergen domain contains four conserved tryptophan residues that form part of the hydrophobic core of this domain [42] (Figure 5). In the *Md*EXPs up to two of these residues occurred and were fully conserved, when the structurally related amino acids phenylalanine and tyrosine are taken into account (Figure 5, Table 1). Although the highly conserved HATFYG motif near the N-terminus is characteristic of EXPA proteins [22], this motif could not be found in the *Md*EXPs. The EXPA and EXPB proteins were distinguished by the presence or absence of short stretches of amino acids at conserved positions at either side of the HFDL motif in the GH45 active site (α- and β-insertions) [16,43]. According to the phylogeny, the *Md*EXPs contained an α-insertion characteristic of EXPAs, but they lacked the four highly conserved N-terminal residues 'GWCN' found in other EXPAs [16]. Of the HFDL motif, only the leucine residue was conserved (Figure 5). However, the long C-terminal extension of *MdEXP2 *was typical for EXLA proteins [22]. Although *Md*EXPs were heterogeneous and divergent, they clearly shared several characteristics of the EXPA protein domains, supporting our phylogenetic results.

**Table 1:** Characteristics (domains and motifs) of the *Micrasterias denticulata *expansin-resembling proteins

Characteristic	MdEXP1	MdEXP2	MdEXP3	MdEXP4
Signal peptide	1-24	1-23	1-19	1-24
GH45 domain	No	39-180	52-211	No
Eight conserved cysteines	Yes	Yes	Yes	Yes
GGACGY motif	GGsCGY	GGsCGf	GGsCGY	GGsCGY
GxxCGxCF/Y motif	Yes	Yes	Yes	Yes
Y/FRRVPC motif	IYAVSC	FTQVPC	VTRVPC	VYAAGC
Catalytic site key residues	No	1 A	1 A	No
DPBB_1 domain	51-132	56-136; 302-385	82-161	51-132
Pollen_allerg_1 domain	144-223	147-226	172-253	No
Four conserved tryptophan (W) residues (* structurally related residues)	2(W) 1(F*) 1(Y*)	2(W) 1(F*) 1(Y*)	2(W) 2(Y*)	No
HATFYG motif (A)	No	No	No	No
α-insertion (A)	Yes	Yes	Yes	Yes
β-insertion (B)	No	No	No	No
HFDL motif (A, B)	Only L	Only L	Only L	Only L
CDRC motif (LA)	No	No	No	No
Long carboxy terminal extension (LA)	No	Yes	No	No

When a domain is present, its position is given (starting from the first methionine). A, unique characteristic of the EXPA family; B, unique characteristic of the EXPB family; LA, unique characteristic of the EXLA family; LB, unique characteristic of the EXLB family

### Subcellular localization of the expansin-resembling *MdEXP2 *and phenotypic changes due to its overexpression

**Figure 8:**
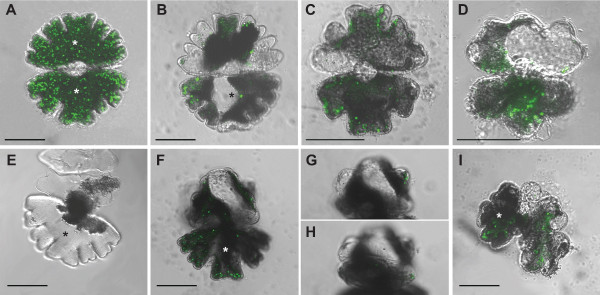
**Phenotypes of *Micrasterias denticulata *cells transiently overexpressing *MdEXP2-GFP *observed by confocal fluorescence microscopy**. Merged transmission light and GFP fluorescence single optical sections (B-I) or projection (A). Initial semicells not formed under *MdEXP2-GFP *overexpression marked by asterisk. (A) Undivided *MdEXP2-GFP *overexpressing cell. (B-I) Phenotypes of *M. denticulata *cells transiently overexpressing *MdEXP2-GFP *arranged according to phenotype severity. (B) Cell line 11. Upper semicell formed after the first cell division, exhibiting stimulated lobe elongation. The lobes are stretched and rounded instead of flattened at their tips. (C-E) Elongation growth is reduced, lateral lobes are fused. (C) Cell line 6. Lower semicell formed after the second, upper semicell after the third cell division. (D) Cell line 7. Lower semicell formed after the first, upper semicell after the second cell division. (E) Cell line 18. Upper semicell resulting from the first cell division, after which the cell died. (F-I) Cell line 13. Loss of growth polarity and planarity upon cell division. (G, H) Other focal sections of (F) showing that there are three growth planes instead of one. (I) Semicells fused upon the second cell division. Scale bar = 50 μm.

**Figure 9:**
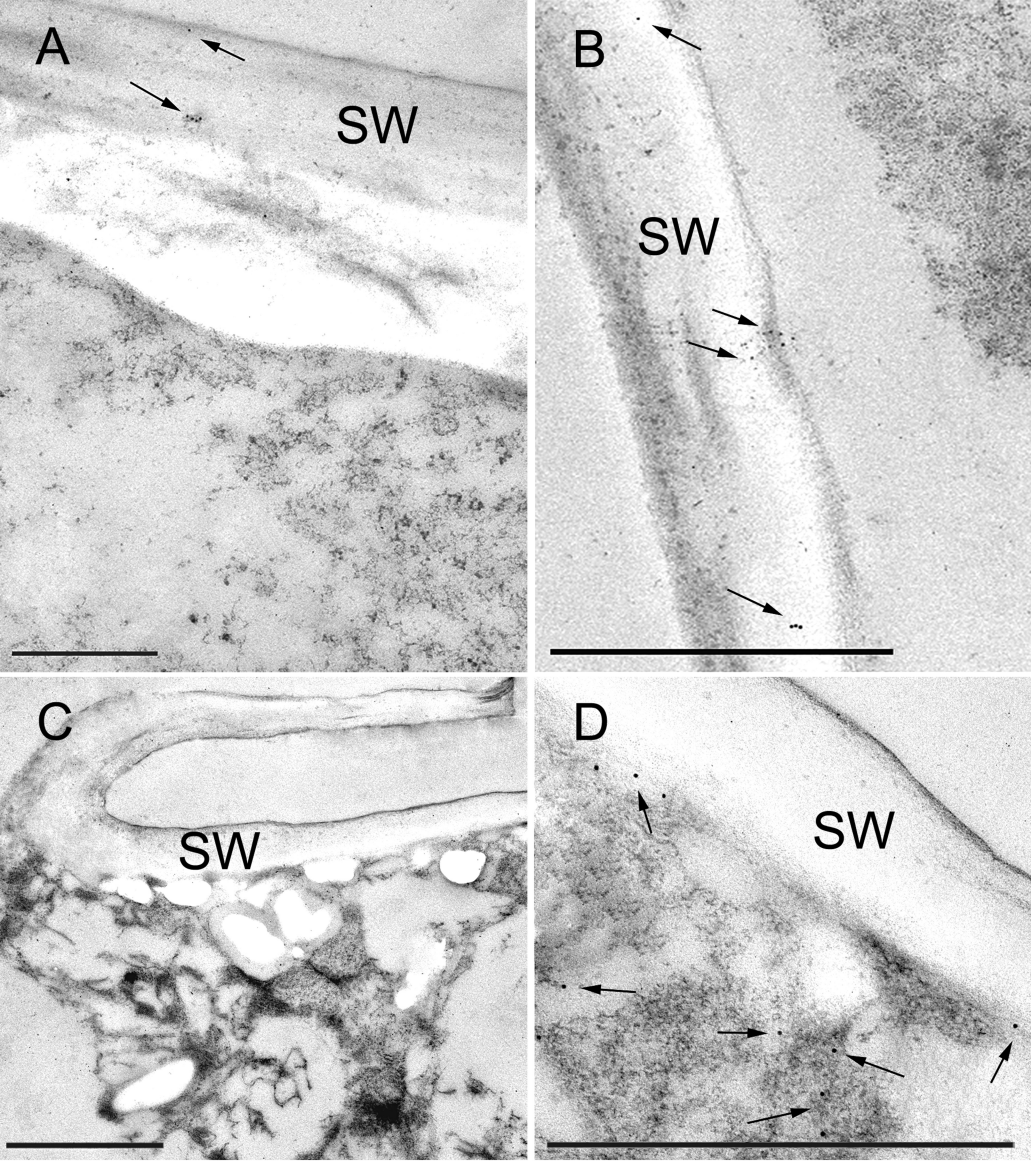
**Immunogold labelling with anti-GFP antibody of high pressure-freeze fixed *Micrasterias denticulata *interphase cells**. (A) and (B) Positive signal present in the secondary cell wall (arrows) and absent from the cytoplasm in *MdEXP2*-*GFP-*overexpressing cells. Detachment of the wall from the cytoplasm is a preparation artefact. (C) and (D) Label present in the cytoplasm and absent from the cell wall in cells overproducing the free GFP. (D) Inset of (C). SW, Secondary cell wall. Scale bar = 1 μm (A, B, D) and 2 μm (C).

**Figure 10:**
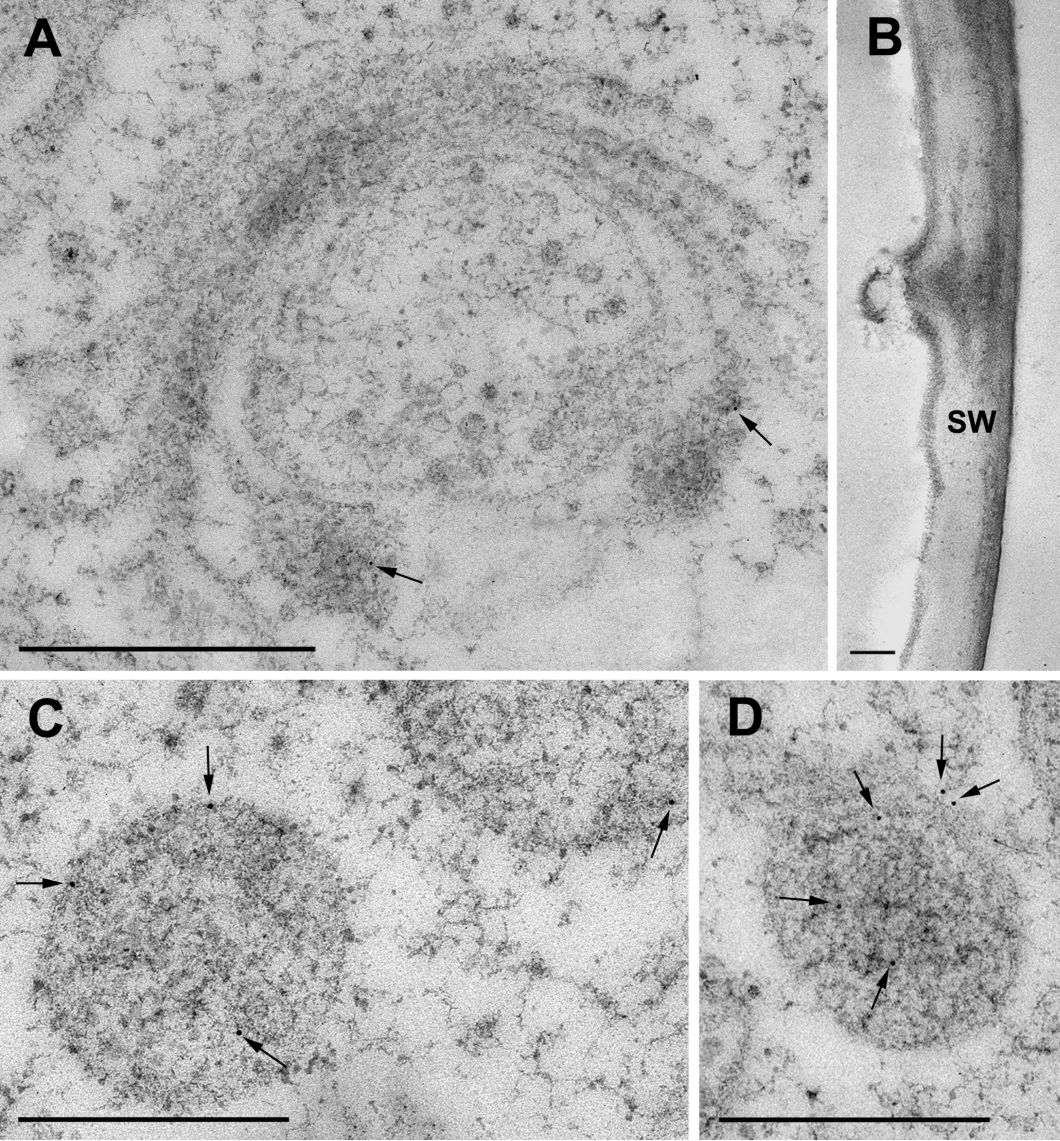
**Immunogold labelling with anti-GFP antibody of high pressure-freeze fixed transiently *MdEXP2-GFP*-overexpressing *Micrasterias denticulata *interphase cells**. (A) No staining of dictyosomal cisternae, fine staining of vesicles attached to the dictyosome (arrows). (B) Control staining with protein A-gold alone. No signal. SW, Secondary cell wall. (C, D) Staining of a mucilage vesicle released into the cytoplasm. Arrows indicate the label. Scale bar = 1 μm.

The ORF of the *M. denticulata *expansin-resembling protein with the highest mRNA levels during cell growth, namely MdEXP2, was cloned into an overexpression vector to allow C-terminal fusions to the green fluorescence protein (GFP) [35]. As observed by confocal laser scanning microscopy of transiently *MdEXP2-GFP-*overexpressing interphase cells, the MdEXP2-GFP fluorescence occurred as motile cytoplasmic dots (Figure 8; Additional file 12) but could not be observed in the secondary cell wall itself, probably because of quenching due to a low apoplast pH [44]. Therefore, *MdEXP2-GFP*-overexpressing interphase cells were processed for transmission electron microscopy (TEM) and stained with GFP antibodies and protein A-gold to investigate whether the MdEXP2-GFP protein localizes into the secondary cell wall. Indeed, a positive signal was observed in the secondary cell wall, albeit not abundantly (Figure 9A,B), probably due to the instability of the GFP protein in this acid compartment [44]. In addition, mucilage vesicles still attached to distal Golgi cisternae (Figure 10A) and some released from the dictyosome (Figure 10C,D) were stained. This immunogold labelling indicated that the punctate pattern of the GFP fluorescence (Figure 8A) could correspond to Golgi-derived mucilage vesicles and that the fusion protein was directed to the wall via the endoplasmic reticulum-Golgi secretory pathway. No staining was observed in experiments for specificity control consisting of sections treated with protein A-gold alone (Figure 10B). In control sections of transgenic cells producing the free GFP, labelling occurred in the cytoplasm and was absent from the cell wall and cell organelles (Figure 9C,D).

Next, 26 independent transient transgenic cells were isolated and further analysed (Additional file 13). A group of cells lost the GFP-fluorescence within a few days and divided, resulting in normal daughter cells, while the majority of the cells died, possibly because of strong *MdEXP2 *overexpression as indicated by their bright GFP fluorescence. However, in eight independent cell lines, a range of phenotypes related to *MdEXP2 *overexpression during cell division and growth could be observed. Line 11 exhibited strong lobe elongation without loss of growth polarity after the first cell division (Figure 8B). The lobes were stretched and rounded instead of flattened at their tips. After the second cell division of line 11 and in all other cases (lines 6, 7, 8, 12, 13, 18, 19), the growth polarity was altered. Line 13 lost its planarity upon cell division and, thus, had the most severe phenotype. New semicells, without the characteristically lobed morphology, but almost without indentations, grew out three-dimensionally. Upon a new cell division of one of the daughter cells, the same phenotype was observed, whereas the newly formed semicells were also fused with each other (Figure 8F-I). In lines 6, 7, 8, 11 (from the second cell division onwards), 12, 18, and 19 axial but not radial elongation was impaired, resulting in semicells with a stunted polar lobe and fused lateral lobes (Figure 8C-E). Sometimes, the second division gave rise to a similar morphology (Figure 8D), but in most cases the phenotype was lost over one to two subsequent generations (Figure 8C). That all phenotypes still had the GFP signal and none of them resulted from control experiments with transgenic cells expressing only the *GFP *[35] suggests that they were related to the expression of the transgene.

## Discussion

Genome-wide expression analysis revealed a role for Rab and SNARE cycles in membrane fusions and for AGP-like proteins in cell pattern establishment. AGPs, differing in composition from land plants, had recently been found to be present in the growing primary cell wall of *Micrasterias *[13]. Our analysis further suggests an involvement of class-III peroxidases, XTH and expansins in cell wall growth. Class-III peroxidases had been considered absent in green algae [45], although a (partial) mRNA occurred in the desmid *Closterium *[46]. Here, a full length algal class-III peroxidase is linked to cell growth. Furthermore, despite their supposed lack of xyloglucans [47], XET activity was found in the streptophyte *Chara *and the chlorophyte *Ulva *[48]. Recently, the (1→3, 1→4)-β-glucan (mixed linked glucan, MLG) has been determined as the main constituent of the secondary cell wall of *Micrasterias *[13] and in this study, the first algal XTH was identified.

The green algae *Valonia *(Chlorophyta) and *Nitella *(Streptophyta) exhibit acid-induced wall extension, but this response is seemingly not mediated by proteins [49,50]. Contrary to the assumption of a land plant specific mechanism [20], four genes with significant similarity to expansins were up-regulated during cell growth of *Micrasterias*, in agreement with a presumed ancient evolutionary origin [16]. Based on significant BLAST similarities with the expansin domains [22], global pairwise alignment and phylogeny, and structural features like the presence of a secretion signal, MdEXP2, MdEXP1, and MdEXP3 are considered expansins, but considerably diverge in gene architecture from embryophytic expansins, as indicated by both domain analysis and phylogeny. These results add to the evidence that expansins are not strongly conserved through evolution [17]. The key residues of the GH45 domain catalytic site and the HFDL motif, which are present in land plants and *Spirogyra*, do not occur in *Micrasterias*. The HFDL motif is present in most groups of plant expansins, but is absent in a few plant EXPA and EXPB proteins [16]. The eight N-terminal cysteines required for protein folding [16] and the four C-terminal tryptophans or related residues involved in cellulose binding [42] are conserved between *Micrasterias, Spirogyra *and land plants and can be considered as key characteristics of plant expansins. The GGxCGY/F and the GxxCGxCF/Y motifs in the GH45 domain are conserved as well. The only constant difference in the conserved amino acid residues in *Micrasterias *when compared to land plants is the occurrence of a serine residue instead of an alanine residue in the GGACGY motif of the GH45 domain. As expansins disrupt noncovalent bonding between cellulose microfibrils and matrix glucans that stick to the microfibril [18], we hypothesize that the characteristics of the *Md*EXPs might be related to the dominant MLG in the secondary cell walls of *Micrasterias *[13] instead of the (1→4)-β-glucan backbone present in dicotyledonous plants. The occurrence of MLG in lichens [51], fungi [52], green algae (*Micrasterias*) [13], horsetails [53], and Poales [54] has been suggested to result from convergent evolution [55], whereas the occurrence of distinct *Md*EXPs might be connected to two different (primary and secondary) cell wall types, implied by their different temporal expression patterns.

Based on the present expansin phylogeny, combined with current hypotheses on the evolution of the closest relatives to land plants [6,56], expansins can fairly be assumed to have evolved before the origin of land plants. However, the unresolved relationships between Embryophytes and the streptophyte lineages Zygnematophyceae, Coleochaetophyceae and Charophyceae [57] hamper a solid reconstruction of expansin gene history. Assuming that the Zygnematophyceae form the closest living relatives to land plants [8], a possible scenario would be that expansins evolved into two lineages (EXPA and EXPB + EXL) in a common ancestor of Embryophytes and Zygnematophyceae (Figure 6B). The apparent lack of EXPB and EXL in *Micrasterias *and *Spirogyra *might be due to gene loss, early in the evolution of the Zygnematophyceae. It should be emphasized however, that the ancient relationships among expansin families are difficult to resolve. Therefore the phylogenetic positions of the green algal expansin-resembling genes should be interpreted with care, hinting at a complete divergence of the plant expansin families within the embryophytic lineage.

Distinct differences in gene architecture between *Micrasterias *and embryophytic expansins have raised the question whether the biochemical functions of *Md*EXPs and embryophytic expansins are similar. To this end, we studied functionally *MdEXP2*, the *MdEXP *with the highest expression levels during growth, through localization and overexpression. A GFP antibody detecting the MdEXP2-GFP fusion protein was used, because the sequence conservation was too low for the available plant expansin antibodies. Unfortunately, currently, because only transient genetic transformation of *Micrasterias *is possible [35], immunoelectron microscopic detection in the growing cell walls is unfeasible. Nevertheless, the ectopically produced protein was targeted to the fully-grown secondary cell wall. In addition, the phenotypic results obtained from its overexpression suggest that MdEXP2 can alter the cell wall shape, but this effect on growth cannot be excluded to result from saturation or blockage of the membrane trafficking of other essential proteins. The phenotypes were remarkably variable, whereby the phenotype severity did not seem to directly correlate with expansin abundance (as inferred from MdEXP2-GFP fluorescence intensity), as reported previously [58-62]. Although a phenotype could be observed corresponding to the expected enhanced wall extensibility due to increased expansin levels [19], the elongation growth impaired in most cases, but not the lateral expansion, resulting in the fusion of the lateral lobes. A number of factors might explain the reduced growth of tomato (*Solanum lycopersicum*) overexpressing an expansin [59]. All together, the growth phase-specific expression, the accumulation in the cell walls, and its overexpression phenotype, allow us to to hypothesize that *MdEXP2 *might have a biochemical function related to that of land plant expansins.

## Conclusions

Our study provides novel data on gene expression during morphogenesis and cell growth in the desmid *Micrasterias denticulata *and adds to our understanding of the evolution of genes involved in cell wall formation in green algae and land plants.

Cell walls have played crucial roles in the colonization of land by plants [63,64]. For a detailed understanding of how cell walls have evolved, cell wall components and cell wall-related genes in land plants and their closest relatives, the streptophyte green algae need to be analyzed comprehensively. Although some cell wall components appear to be adaptations of land plants, cell wall evolution after the colonization of land is seemingly characterized by the elaboration of a pre-existing set of genes and polysaccharides rather than by substantial innovations [65-68]. The data add to the growing body of evidence that the evolutionary origins of many cell wall components and regulating genes in embryophytes antedate the colonization of land.

## Methods

### Culture conditions, synchronization and sampling

A clonal *Micrasterias denticulata *culture was grown in twofold diluted Desmidiaceae medium [69] at 23°C and 120-140 μmol photons.m^-2^.s^-1 ^under a 14-h light/10-h dark regime.

Two independent cultures were synchronized by replacing the growth medium of a stationary culture, diluting the density, and extending the light period to 24 h. Cells were sampled from synchronized cultures for RNA extraction at five consecutive time points during growth (T1 to T5) that were chosen to include for each time point a different proportion of cells at different morphogenetic stages (bulge, lobe, and doublet stage) (Figure 1C,D). Two independent stationary cultures served as control samples. Cells were concentrated by centrifugation for 1 min at 4°C and 4000 rpm and washed with phosphate buffered saline (PBS). Cell pellets were snap-frozen in liquid nitrogen and stored at -70°C.

### RNA extraction and cDNA-AFLP analysis

Total RNA was isolated from approximately 80,000 frozen cells at each time point as described [70] with slight modifications. Instead of β-mercaptoethanol, 2 M stock solution of the anti-RNase agent dithiothreitol was added to the extraction buffer to a final concentration of 50 mM [71]. Cells were disrupted and homogenized in a bead mill (Retsch) (5 min at frequency 30 s^-1^) with silicone-carbide sharp particles (Biospec Products) after the cell pellet had been thawed and suspended in the extraction buffer. Phytopure resin (GE-Healthcare) was added during the first chloroform:isoamylalcohol extraction to eliminate mucilage contamination of the RNA [72]. RNA samples were controlled qualitatively with the RNA 6000 Nano kit of the Bioanalyzer 2100 (Agilent Technologies) and quantified with the ND-1000 UV-Vis Spectrophotometer (Nanodrop).

Starting from 2 μg of total RNA, cDNA synthesis and cDNA-AFLP analysis with *Bst*YI and *Mse*I as restriction enzymes were done according to the procedures as described [28]. For the selective amplification, *Bst*YI + C/T + 1/*Mse*I + 2 primer pairs were used, resulting in 128 primer combinations. The cDNA-AFLP fingerprints were visualized with an autoradiography platform (PhosphorImager 445 SI; Molecular Dynamics).

Scanned gel images were quantitatively analyzed with the AFLP-QuantarPro image analysis software (Keygene N.V.). Expression values per gene were normalized for replicate effects by subtracting the replicate mean value (Additional file 2). Average linkage hierarchical clustering with the TMeV v4 software (http://www.tm4.org) and adaptive quality-based clustering (minimum two tags in a cluster, 0.95 significance level) [73] of the normalized expression data were carried out.

To assess the effect of the various cell division stages (T1-T5) on the gene expression during synchronized growth, a linear regression model of the form *y *= *μ *+ *rep *+ *time *+ *ε *was fitted to the data, where *y *represents the raw expression values, *rep *and *time *the fixed replicate and time effects, respectively, and ε the random error. For all TDFs, a *F*-statistic was calculated, *P*-values were assigned to the main term *time *and subsequently transformed into false discovery rates, and *Q*-values [74] (Additional file 2). Besides the TDFs with a significant (*Q*<0.05) differential expression across the five time points, TDFs that were clearly absent in the stationary cultures but present during the synchronized growth, were excised from the dried gels, irrespective the significance of their differential expression across the five stages, followed by amplification and subsequent sequencing [28].

The TDFs were designated by *Md *(for *M. denticulata*) followed by a number corresponding to the AFLP fragment. After mutual alignment of the sequences, only the longest one of a group of identical sequences was retained, except when the TDFs displayed different expression profiles. Each sequence was identified by a similarity search against the public databases with the Blast2GO v1.7.2 program (http://www.Blast2GO.de) [75]. In addition to hits displaying an E-value < 1.E-03, hits with E-values between 1.E + 00 and 1.E-03 and a similarity > 50% were retained for further analysis.

### Real-time qRT-PCR assay

Primers (Additional file 14) were designed with the Beacon Designer 7.0 (PREMIER Biosoft International) and the Oligo PerfectTM Designer (Invitrogen). Isolated RNA was treated with DNaseI (GE-Healthcare). An aliquot of 1 μg of total RNA from each sample was used for cDNA synthesis. The reverse transcription was carried out with oligo-dT primers and the SuperscriptTM II reverse transcriptase (Invitrogen) according to the manufacturer's instructions.

A set of reference genes was selected, based on their constitutive expression pattern during morphogenesis, to serve as a normalization factor in quantitative reverse-transcription-(qRT)-PCR analysis. Their expression stability (*M*) was analyzed with the geNormTM program [76]. Among 10 constitutively expressed TDFs, *Md*0789 (similar to a reticulon of *Arabidopsis thaliana*) and *Md*1473 (similar to peroxiredoxin 6 of Norway rat [*Rattus norvegicus*]) (*M *value = 0.421 and 0.489, respectively) were the two most stably expressed genes, followed by *Md*0386 (similar to the unknown gene of *A. thaliana *at5g13390 t22n19_40). To determine the number of internal control genes necessary for reliable data normalization, the pairwise variation value between two sequential normalization factors V_2/3 _was calculated with geNormTM and turned out to be 0.151 under our experimental conditions, slightly higher than the cut-off value of 0.15. The inclusion of a fourth internal control gene resulted in an increase of the pairwise variation, yielding a V_3/4 _value of 0.129. As a result, the use of the two or three most stably expressed genes was considered to be sufficient for reliable data normalization [76]. PCR fragments were amplified in triplicate on the LightcyclerTM 480 (Roche Applied Science) platform with SYBRTM Green QPCR Master Mix (Stratagene), according to the manufacturer's instructions with cycling conditions of 10 min preincubation at 95°C and 45 cycles at 95°C for 10 s, 58°C for 15 s, and 72°C for 15 s. Amplicon dissociation curves were recorded by heating from 65°C to 95°C. qBaseTM [77] was used for relative quantification.

### RACE PCRs, protein domain identification, sequence alignment and phylogenetic analyses

The ends of the cDNAs were obtained by rapid amplification of cDNA ends (RACE) PCRs with plasmid DNA from a cDNA library of growth-synchronized *M. denticulata *(purchased from Invitrogen) as template. For *MdEXP1, MdEXP3*, and *Md*0434 only 5' RACE PCR was done, because the TDF contained the stopcodon and a part of the 3' untranslated region. For *Md*4341, *MdXTH1, MdEXP2*, and *MdEXP4 *both 5' RACE and 3' RACE were necessary. Gene-specific primers were designed with the eprimer3 program [78] and used in combination with vector-specific (pDONR222.1) primers (Additional file 15) in a PCR consisting of 1 min pre-incubation at 95°C and 30 cycles at 95°C for 30 s, 54°C for 30 s and 72°C for 2 min 30 s, followed by 1 cycle at 72°C for 5 min.

Protein domains in the ORF sequences were identified with the SMART tool (http://smart.embl-heidelberg.de/) [79,80]. Signal sequences were confirmed with the SignalP 3.0 Server (http://www.cbs.dtu.dk/services/SignalP) [81,82] and iPSORT prediction (http://ipsort.hgc.jp/) [83].

Similar sequences were retrieved from GenBank (http://www.ncbi.nlm.nih.gov) using protein BLAST and tblastx [84] (Additional file 16). The sequences were aligned using MUSCLE [85]. To remove signal peptides and C-terminal extensions, the alignment was trimmed from a conserved tryptophan near the N-terminus to a conserved phenylalanine near the C-terminus [17].

Two sets of alignments were considered for the phylogenetic analyses. The first dataset consisted of the four *M. denticulata *expansin-resembling proteins (*Md*EXPs), 26 land plant expansins representing the 17 orthologous clades within the four land plant expansin families [22], and six EST sequences of the streptophyte green alga *Spirogyra pratensis *that showed significant similarity to land plant expansins [27]. Five expansin-like sequences of the social amoeba, *Dictyostelium discoideum*, were selected as outgroup based on their inferred relationship with land plant expansins [16,86]. This alignment was 227 amino acids long (Additional file 17). The second dataset included all sequences of the first alignment plus nine putative expansin genes found in four species of Chlorophyta (the sister clade of the Streptophyta) (322 amino acids long; Additional file 18) and was used to assess the phylogenetic position of other putative expansin sequences of green algae.

Models of protein evolution were selected with ProtTest 1.4 [87]. Maximum likelihood (ML) and Bayesian phylogenetic inference (BI) were analyzed under a WAG model of amino acid substitution with among site rate heterogeneity (gamma distribution with eight categories) for all datasets with PhyML v2.4.4 [88] and non-parametric bootstrapping (1000 replicates) to assess statistical support of internal branches with MrBayes 3.1.2 [89], respectively. Two parallel runs, each consisting of four incrementally heated chains were run for 2 x10^6 ^generations, sampling every 1000 generations. Convergence of log-likelihoods was assessed in Tracer v1.4 [90]. A burn-in sample of 500 trees (well beyond the point at which convergence of parameter estimates had taken place) was removed before the majority rule consensus trees were constructed.

### Overexpression of *MdEXP2 *and microscopy

The ORF of *MdEXP2 *was cloned into the *Spe*I restriction site (ACTAGT) of the vector pSA405A under the control of the chlorophyll *a/b*-binding protein encoding gene of the desmid *Closterium *and was C-terminally fused to the green fluorescence protein gene (*GFP*) [35]. Primers were: forward primer 5'-ATGACTAGTATGAAAATCGGCATAATCCA-3' and reverse primer 5'-GGAACTAGTTAGGCACCCATTAACGGC-3'. The PCR was 2 min preincubation at 94°C and 5 cycles at 94°C for 45 s, 45°C for 45 s, and 68°C for 3 min, followed by 30 cycles at 94°C for 45 s, 55°C for 45 s, and 68°C for 2 min, and by 1 cycle at 72°C for 5 min. The recombined plasmid was introduced into *M. denticulata *by microparticle bombardment [35].

For confocal microscopy, a 100M microscope (Zeiss) was used, equipped with the LSM510 software version 3.2. Samples were scanned with a 20x (numerical aperture of 0.5) and a 63x water corrected objective (numerical aperture of 1.2). GFP fluorescence was visualized with argon laser illumination at 488 nm and a 500 to 530 nm band emission filter.

For transmission electron microscopy (TEM), a GFP antibody (Rb, (ab6556) Abcam) compatible protocol was followed to prepare the samples. *MdEXP2-GFP*-overexpressing *M. denticulata *cells were embedded in a yeast paste in a membrane carrier (Leica) and frozen immediately in a high-pressure freezer (EM PACT; Leica Microsystems). Freeze substitution was carried out in a Leica EM AFS instrument. Samples were infiltrated at 4°C stepwise in LR-White, hard grade (London Resin Company Ltd.) and embedded in capsules. Ultrathin sections of gold interference color were cut with an EM UC6 ultramicrotome (Leica) and collected on formvar-coated copper mesh grids. Grids were floated on blocking solution followed by incubation in a 1:25 and 1:10 dilution (in 1% bovine serum albumin in PBS) of primary antibodies (GFP antibody, (Rb, (ab6556) Abcam) for 60 min. The grids were labelled with protein A-10-nm gold (PAG10nm) (Cell Biology Department, Utrecht University). Control experiments consisted of treating sections with PAG10nm alone. Sections were post-stained in an automatic contrasting instrument (EM AC20; Leica Microsystems GmbH) for 30 min in uranyl acetate at 20°C and for 7 min in lead stain at 20°C. Grids were viewed with a 1010 transmission electron microscope (JEOL) operating at 80 kV.

Newly obtained sequence data were deposited in GenBank; the transcript derived fragments under accession numbers HE578289 to HE578716, the reference genes under accession numbers HE580226 to HE580228, and the open reading frames under accession numbers HE578717 to HE578726.

## Authors' contributions

KV carried out the molecular genetic studies and drafted the manuscript. MJJH participated in the real-time qRT-PCR assay and genetic transformation. RDR carried out the immunoelectron microscopy. MV designed the cDNA AFLP study and performed the statistical analyses. FL carried out the phylogenetic analysis. JP and AG participated in the RACE PCRs. UL-M participated in the synchronization and RNA-extraction and in the interpretation of the data. JG and LDV participated in the design of the experiments. DI and WV conceived and supervised the study. WV, FL, LDV and MH helped to draft the manuscript. All authors read and approved the final manuscript.

## Supplementary Material

Additional file 1 **Distribution of morphogenetic stages in the RNA samples used for cDNA-AFLP, replication 2, and real-time qRT-PCR**. Click here for file

Additional file 2 **Expression values of all scored TDFs**. Click here for file

Additional file 3 **Similarities of cDNA-AFLP fragments to database sequences**. Click here for file

Additional file 4 **Raw real-time qRT-PCR expression values**. Click here for file

Additional file 5 **Comparison of the expression profiles of selected TDFs obtained by cDNA-AFLP and qRT-PCR for the samples of replication 2**. Click here for file

Additional file 6 **Full-length deduced amino acid sequence of *Md*4341 aligned with its relevant BLAST hits**. Click here for file

Additional file 7 **Full-length deduced amino acid sequence of MdXTH1 (*Md*0888) aligned with its relevant BLAST hits**. Click here for file

Additional file 8 **Full-length deduced amino acid sequence of *Md*0434 aligned with its relevant BLAST hits**. Click here for file

Additional file 9 **Characteristics of the expansin-resembling genes from *Micrasterias denticulata***. Click here for file

Additional file 10 **Unrooted maximum likelihood phylogeny showing the relationship of putative chlorophytan expansin sequences (with significant similarity to plant expansins in tblastx searches) with the plant expansin gene family**. Click here for file

Additional file 11 **Protein BLAST alignment of MdEXP2 with its best hit, showing the expansin-like C-terminal extension**. Click here for file

Additional file 12 **Confocal GFP fluorescence time lapse images (30 s apart) illustrating the motility of the MdEXP2-GFP containing intracellular compartments**. Click here for file

Additional file 13 **Features of transgenic cell lines overexpressing the *MdEXP2*-*GFP *fusion gene**. Click here for file

Additional file 14 **Primer sequences of selected *Micrasterias denticulata *TDFs used for real-time qRT-PCR assay**. Click here for file

Additional file 15 **Primers used for RACE PCR and cloning**. Click here for file

Additional file 16 **Accession numbers of the sequences used to construct the phylogenetic trees and additional *Physcomitrella patens *sequences**. Click here for file

Additional file 17 **Nexus file with the MUSCLE alignment used in this study for phylogenetic analyses of expansins**. Click here for file

Additional file 18 **Nexus file of the MUSCLE alignment used in this study for phylogenetic analyses of expansins including sequences of the Chlorophyta**. Click here for file
