# Supplementary material for: Transcriptional analysis of cell growth and morphogenesis in the unicellular green alga Micrasterias (Streptophyta), with emphasis on the role of expansin
Source: BMC Plant Biol. 2011 Sep 25;11:128. doi: 10.1186/1471-2229-11-128 (PMC3191482; doi:10.1186/1471-2229-11-128)
Supplement: Additional file 1 — Distribution of morphogenetic stages in the RNA samples used for cDNA-AFLP, replication 2, and real-time qRT-PCR. [file 1471-2229-11-128-S1.PDF]

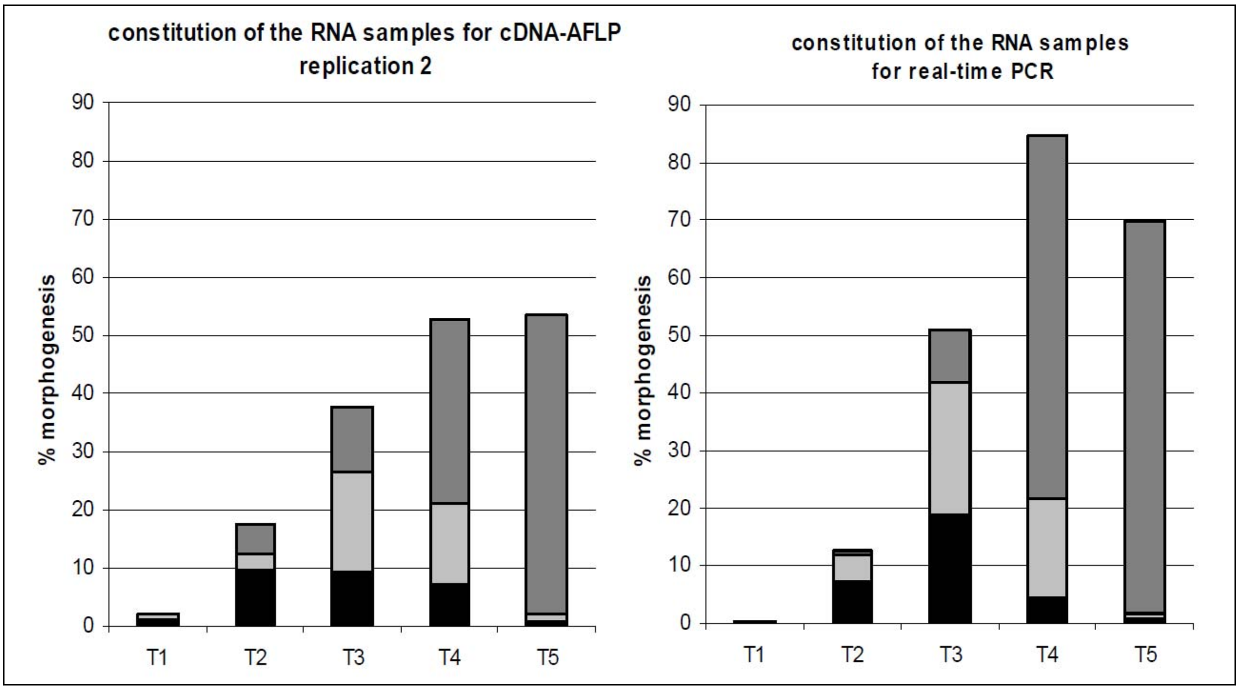

Additional file 1. Distribution of morphogenetic stages in the RNA samples used for cDNA-AFLP, replication 2, and real-time qRT-PCR. Black bars: bulge-stage cells, light grey bars: lobe-stage cells, dark gray bars: doublet cells.
