# Supplementary material for: Transcriptional analysis of cell growth and morphogenesis in the unicellular green alga Micrasterias (Streptophyta), with emphasis on the role of expansin
Source: BMC Plant Biol. 2011 Sep 25;11:128. doi: 10.1186/1471-2229-11-128 (PMC3191482; doi:10.1186/1471-2229-11-128)
Supplement: Additional file 3 — Similarities of cDNA-AFLP fragments to database sequences. [file 1471-2229-11-128-S3.PDF]

Additional file 3. Similarities of cDNA-AFLP fragments (transcript derived fragments, TDFs) to database sequences.

TDF	length (bp)	sequence similarity	E-value	similarity (%)	expression
Cell division					
Md3391	398	CAL50518.1: cyclin B [*Ostreococcus tauri*]	1,00E-06	64	C1A
Md3159	199	AAP73784.1: cyclin-dependent kinase CDKB [*Populus tremula x Populus tremuloides*]	1,00E-08	80	C1A
Cell wall metabolism					
Md3598	449	CAI11451.1: α-1,6-xylosyltransferase [*Gossypium raimondii*]	1,00E-41	75	C1A
Md1480	187	NP_826382.1: α-L-arabinofuranosidase B [*Clostridium thermocellum* DSM 2360]	1,00E-04	51	C3
Md3604	298	AAF17570.1: α-expansin 1 [*Marsilea quadrifolia*]	1,00E-05	48	C3
Md1418	501	AAN08121.1: α-expansin 5 [*Physcomitrella patens*]	1,00E-06	52	C4
Md2820	157	AAM73779.1: β-expansin 15 [*Oryza sativa*]	1,00E-04	68	C4
Md3497	403	AAK56127.1: β-expansin 4 [*Zea mays*]	1,00E-04	53	C3
Md0559	428	NP_001031975.1: β-glucosidase [*Arabidopsis thaliana*]	1,00E-25	76	C4
Md4113	224	ZP_06634597.1: cell wall-associated hydrolase [*Aggregatibacter aphrophilus* NJ8700]	1,00E-11	85	C1b
Md0757	312	AAQ63935.1: cellulose synthase [*Pinus radiata*]	1,00E-06	84	C3
Md3668	212	AAM83096.1: cellulose synthase catalytic subunit [*Mesotaenium caldariorum*]	1,00E-30	90	C3
Md2838	480	ACV31215.1: cellulose synthase-like C4 [*Hordeum vulgare* subsp.*vulgare*]	1,00E-06	75	C3
Md0493	287	ACJ11760.1: class III peroxidase [*Gossypium hirsutum*]	1,00E-16	39	C3
Md0434	409	ACJ11764.1: class III peroxidase 17 [*Gossypium hirsutum*]	1,00E-17	65	C1A
Md2144	337	NP_198941.1: exostosin family protein [*Arabidopsis thaliana*]	1,00E-06	58	C1A
Md0450	153	NP_201028: exostosin family protein [*Arabidopsis thaliana*]	1,00E-02	50	C3
Md1114	571	ABA99677.1: exostosin family protein [*Oryza sativa*]	1,00E-16	54	C3
Md3053	294	NP_198236.1: GDP-mannose 3,5-epimerase [*Arabidopsis thaliana*]	1,00E-45	88	C3
Md0808	509	ZP_05532329.1: glycoside hydrolase family protein [*Streptomyces viridochromogenes* DSM 40736]	1,00E-09	48	C5
Md0257	665	NP_001007339.1: glycosyltransferase 8 [*Danio rerio*]	1,00E-11	61	C3
Md1709	678	NP_197459.1: glyoxal oxidase [*Arabidopsis thaliana*]	1,00E-23	50	C3
Md3495	420	XP_001878494.1: glyoxal oxidase [*Laccaria bicolor* S238N-H82]	1,00E-13	59	C1A
Md0606	389	XP_001937507.1: glyoxal oxidase [*Pyrenophora tritici-repentis* Pt-1C-BFP]	1,00E-08	54	C3
Md4415	427	NP_001151351.1: pectinesterase [*Zea mays*]	1,00E-12	53	C1A
Md2842	310	NP_001105405.1: phosphoglucomutase 2 [*Zea mays*]	1,00E-15	79	C1A
Md3500	406	AAD22651.1: putative polygalacturonidase [*Arabidopsis thaliana*]	1,00E-11	54	C5
Md1089	127	NP_177978.1: rhamnose synthase [*Gossypium hirsutum*]	1,00E-12	88	C1b
Md1739	160	ABR15472.1: UDP-glucose pyrophosphorylase [*Pinus taeda*]	1,00E-15	79	C3
Md2333	213	ACL80329.1: UDP-glucose pyrophosphorylase [*Saccharum officinarum*]	1,00E-04	71	C1b
Md2565	482	ACL80329.1: UDP-glucose pyrophosphorylase [*Saccharum officinarum*]	1,00E-04	71	C3
Md0888	440	ACD03231.1: xyloglucan endotransglucosylase/hydrolase 7 [*Malus* x *domestica*]	1,00E-22	53	C3
Cytoskeleton-dependent intracellular transport					
Md0670	422	BY86654.1: beta-tubulin 2 [*Gossypium hirsutum*]	1,00E-40	91	C1A
Md1475	419	BAF46897.1: chloroplast unusual positioning 1A [*Physcomitrella patens*]	1,00E-17	76	C1b
Md1559	359	BAF46897.1: chloroplast unusual positioning 1A [*Physcomitrella patens*]	1,00E-11	84	C3
Md3257	348	AAC49901.1: kinesin-like calmodulin-binding protein [*Arabidopsis thaliana*]	1,00E-05	55	C3
DNA replication					
Md3300	199	AAN87547.1: ribonucleotide reductase [*Glycine max*]	1,00E-15	80	C2
Md0286	199	AAA16231.1: SAICAR synthetase [*Arabidopsis thaliana*]	1,00E-26	92	C4
Fatty acid metabolic process					
Md2839	363	YP_001165955.1: carboxylesterase, type B [*Novosphingobium aromaticivorans* DSM 12444]	1,00E-17	58	C3
Md4413	466	AAP74371.1: fatty acid elongase 1 [*Marchantia polymorpha*]	1,00E-67	91	C3
Md1770	340	NP_177268.1: GDSL-motif lipase/hydrolase 6; carboxylic ester hydrolase	1,00E-10	62	C3

		[*Arabidopsis thaliana*]			
Md1710	688	NP_001149139.1: inositolphosphorylceramide-B C-26 hydroxylase [*Zea mays*]	1,00E-39	64	C3
Md1182	217	BAD08129.1: putative GDSL-motif lipase/hydrolase protein [*Oryza sativa*]	1,00E-07	62	C2
Md0900	203	XP_002722880: putative monoacylglycerol O-acyltransferase 3-like [*Oryctolagus cuniculus*]	1,00E-02	55	C3
Md0382	618	CAB81548.2: putative proline-rich protein APG isolog [*Cicer arietinum*]	1,00E-16	48	C5
Md3533	129	NP_179223.1: very-long-chain fatty acid condensing enzyme [*Arabidopsis thaliana*]	1,00E-06	80	C3
Generation of energy					
Md1066	470	XP_001754045.1: ATP-binding cassette transporter, subfamily G, member 15, group PDR protein PpABCG15 [*Physcomitrella patens* subsp. *patens*]	1,00E-29	77	C5
Md3079	139	CAC80388.1: glyceraldehyde-3-phosphate dehydrogenase [*Marchantia polymorpha*]	1,00E-02	71	C1A
Md1411	133	AAM64828.1: inorganic pyrophosphatase [*Arabidopsis thaliana*]	1,00E-11	81	C3
Md0916	422	NP_196527.1: inorganic pyrophosphatase [*Arabidopsis thaliana*]	1,00E-38	78	C4
Md2837	499	CAM76812.1: phosphoenolpyruvate carboxylase [*Magnetospirillum gryphiswaldense* MSR-1]	1,00E-41	67	C3
Membrane docking					
Md0547	162	YP_001273684: adhesin-like protein [*Methanobrevibacter smithii* ATCC 35061]	1,00E-03	57	C3
Md1560	327	AAN12943.1: putative binding protein; AAA-superfamily ATPase [*Arabidopsis thaliana*]	1,00E-05	51	C3
Md1852	326	AAR13228.1: Rab family GTPase Rab8 [*Fucus distichus*]	1,00E-11	60	C1A
Md0818	245	CAM97308: Rab GDP dissociation inhibitor [*Solanum lycopersicum*]	1,00E-03	91	C1A
Md1404	289	NP_001149760.1: syntaxin 32 [*Zea mays*]	1,00E-19	92	C3
Membrane protein					
Md1409	119	AAA68425.1: chlorophyll a/b-binding protein F3 [*Polystichum munitum*]	1,00E-05	92	C1A
Md0276	329	NP_001148205.1: membrane protein [*Zea mays*]	1,00E-15	64	C5
Md4071	196	XP_001508401.1: predicted: similar to phosphatidylinositol glycan anchor biosynthesis, class F, partial [*Ornithorhynchus anatinus*]	1,00E-03	71	C1A
Md4341	216	XP_001508401.1: predicted: similar to phosphatidylinositol glycan anchor biosynthesis, class F, partial [*Ornithorhynchus anatinus*]	1,00E-04	72	C4
Photosynthesis					
Md1931	232	CAA43907.1: chlorophyll a/b-binding protein [*Pinus thunbergii*]	1,00E-29	92	C1A
Md3781	199	XP_001691223.1: low-CO2 inducible protein [*Chlamydomonas reinhardtii*]	1,00E-05	66	unclustered
Md1217	433	NP_566810.1: plastocyanin-like domain-containing protein [*Arabidopsis thaliana*]	1,00E-05	59	C3
Md1576	106	XP_001766961: PsaH photosystem I reaction center subunit [*Physcomitrella patens* subsp. *patens*]	1,00E-03	75	C1b
Md1344	454	AAA33036.1: ribulose 1,5-bisphosphate carboxylase/oxygenase small subunit [*Mesembryanthemum crystallinum*]	1,00E-51	85	unclustered
Protein metabolic process					
Md0758	327	NP_850566.1: Aha1 domain-containing protein [*Arabidopsis thaliana*]	1,00E-10	91	C3
Md3988	491	YP_003321515.1: Amidase [*Sphaerobacter thermophilus* DSM 20745]	1,00E-19	56	C4
Md0938	203	NP_850663.1: asparagine synthase [*Arabidopsis thaliana*]	1,00E-05	92	C5
Md2541	349	NP_001150767.1: chaperone protein dnaJ 6 [*Zea mays*]	1,00E-26	71	C2
Md1950	100	AAC14026.1: chaperonin 10 [*Arabidopsis thaliana*]	1,00E-02	85	unclustered
Md4521	377	NP_001084790: E3 ubiquitin-protein ligase CBL-B-B [*Xenopus laevis*]	1,00E-02	48	C4
Md0268	369	ABA98760.1: hAT family dimerisation domain containing protein [*Oryza sativa*]	1,00E-03	69	C3
Md3122	136	CAA30018.1: heat shock protein 70 [*Petunia x hybrida*]	1,00E-14	100	unclustered
Md0896	241	XP_001419938.1: heat shock protein 90, cytosolic [*Ostreococcus lucimarinus*]	1,00E-04	82	C5
Md0960	492	ABR25534.1: peptidyl-prolyl cis-trans isomerase [*Oryza sativa*]	1,00E-13	89	C3
Md3870	253	NP_567110.1: zinc finger (c3hc4-type ring finger) family protein [*Arabidopsis thaliana*]	1,00E-12	82	C3
Md1049	261	NP_176427.1: zinc finger (c3hc4-type ring finger) family protein [*Arabidopsis thaliana*]	1,00E-09	65	C5
Regulation of transcription					
Md3264	176	NP_999706.1: histone H2B.1 [*Strongylocentrotus purpuratus*]	1,00E-18	98	C1A
Md2093	364	NP_196747.1: NLI interacting factor (NIF) family protein [*Arabidopsis thaliana*]	1,00E-32	82	C3
Md0850	331	YP_636383.1: putative maturase [*Staurastrum punctulatum*]	1,00E-21	83	C3

Md3355	244	XP_548653.2: similar to double homeobox 4 [*Canis familiaris*]	1,00E-02	71	C3
Md3558	340	XP_001509199.1: similar to high mobility group 1 protein [*Ornithorhynchus anatinus*]	1,00E-07	58	C1A
Md3934	308	ZP_01719535.1: tetratricopeptide repeat protein [*Algoriphagus* sp. PR1]	1,00E-05	53	C3
Signal transduction					
Md1976	428	NP_001147487.1: protein kinase [*Zea mays*]	1,00E-08	60	C3
Md0683	141	ACH85192.1: calcium-dependent protein kinase CDPK5 [*Nicotiana tabacum*]	1,00E-10	82	unclustered
Md3678	198	ACM89604.1: leucine rich repeat protein [*Glycine max*]	1,00E-03	63	C5
Md1340	151	XP_712579.1: putative protein kinase [*Candida albicans* SC5314]	1,00E-06	61	C5
Md2196	438	XP_712579.1: putative protein kinase [*Candida albicans* SC5314]	1,00E-06	61	C5
Md1771	373	BAF79965.1: receptor-like kinase [*Closterium ehrenbergii*]	1,00E-04	52	C3
Md1643	301	BAF79969.1: receptor-like kinase [*Closterium ehrenbergii*]	1,00E-21	70	C3
Md0874	112	EEY56882.1: SCP-like extracellular protein [*Phytophthora infestans* T30-4]	1,00E-02	63	C3
Translation					
Md2915	252	NP_001105477.1: 40S ribosomal protein S21 [*Zea mays*]	1,00E-25	87	C1A
Md1078	200	NP_175314.1: 40S ribosomal protein S7 [*Arabidopsis thaliana*]	1,00E-08	93	C4
Md1327	325	ABD74515.1: ribosomal protein [*Pteris vittata*]	1,00E-27	87	C5
Md1165	117	YP_636418.1: ribosomal protein L23 [*Staurastrum punctulatum*]	1,00E-08	100	unclustered
Transmembrane transporter					
Md0186	175	YP_002944621.1: Extracellular ligand-binding receptor [*Variovorax paradoxus* S110]	1,00E-21	94	unclustered
Md3108	313	AAF86908.1: glucose-6P/phosphate translocator [*Mesembryanthemum crystallinum*]	1,00E-19	77	C1A
Md2939	249	AAR83907.1: heavy metal transporter MTP1 [*Noccaea caerulescens*]	1,00E-16	76	C1A
Md1612	170	ZP_03275620.1: Na-Ca exchanger/integrin-beta4 [*Arthrospira maxima* CS-328]	1,00E-02	50	C3
Md3104	176	ABK91091.1: putative glucose-6-phosphate translocator [*Sorghum bicolor*]	1,00E-20	82	C2
Md0171	518	BAF00815.1: putative multispanning membrane protein [*Arabidopsis thaliana*]	1,00E-59	94	C3
Md3594	694	Q0JAI9.1: putative secretory carrier membrane protein [*Oryza sativa*]	1,00E-41	68	C1A
Md1891	260	NP_199351.1: sodium solute symporter family protein [*Arabidopsis thaliana*]	1,00E-13	69	C3
Md1499	275	YP_136370.1: sodium-solute symporter, putative [*Haloarcula marismortui* ATCC 43049]	1,00E-04	55	C5
Md0487	378	ABB87132.1: translocon-associated protein beta family protein [*Solanum tuberosum*]	1,00E-16	65	C1A
Md3769	336	NP_001148367.1: transmembrane 9 superfamily protein member 1 [*Zea mays*]	1,00E-06	57	C1A
Md0329	478	NP_564133.1: transporter-related [*Arabidopsis thaliana*]	1,00E-40	73	C1A
Unknown					
Md2397	522	AAM61029.1: ankyrin-like protein [*Arabidopsis thaliana*]	1,00E-23	60	C2
Md1425	243	XP_001760143.1: predicted protein [*Physcomitrella patens* subsp. *patens*]	1,00E-09	83	unclustered
Md0677	160	ABK23825.1: unknown [*Picea sitchensis*]	1,00E-18	86	C1A
Md4542	178	XP_002484510.1: hypothetical protein TSTA_040370 [*Talaromyces stipitatus* ATCC 10500]	1,00E-07	96	C1A
Md3859	419	XP_001372919.1: hypothetical protein [*Monodelphis domestica*]	1,00E-02	36	C3
Md2940	344	EEC84822.1: hypothetical protein OsI_31909 [*Oryza sativa* Indica Group]	1,00E-02	54	C5
Md3354	186	CBA31922.1: hypothetical protein [*Curvibacter* putative symbiont of *Hydra magnipapillata*]	1,00E-27	98	C1A
Md2999	147	T19530: hypothetical protein C27H2.1 [*Caenorhabditis elegans*]	1,00E-03	60	C3
Md3087	119	XP_002649059.1: hypothetical protein CBG21992 [*Caenorhabditis briggsae*]	1,00E+00	68	C3
Md2765	313	EDO61937.1: hypothetical protein CLOLEP_01448 [*Clostridium leptum* DSM 753]	1,00E-15	86	C3
Md2728	233	T21438: hypothetical protein F26H9.3 [*Caenorhabditis elegans*]	1,00E-05	56	C3
Md2731	207	ACL54757.1: unknown [*Zea mays*]	1,00E-03	78	C1A
Md4395	181	CAP41015.1: hypothetical protein [*Bordetella petrii*]	1,00E-14	86	C1b
Md4001	248	CBA31922.1: hypothetical protein [*Curvibacter* putative symbiont of *Hydra magnipapillata*]	1,00E-37	93	unclustered
Md0671	385	XP_002178933.1: predicted protein [*Phaeodactylum tricornutum* CCAP 1055/1]	1,00E-04	55	C5
Md1132	242	YP_003012835.1: hypothetical protein Pjdr2_4120 [*Paenibacillus* sp. JDR-2]	1,00E-02	56	C5
Md1650	215	YP_827473.1: phosphate-responsive 1 family protein [*Solibacter usitatus*]	1,00E-02	56	C4

Md2913	354	XP_001422669.1: predicted protein [*Ostreococcus lucimarinus*]	1,00E-02	48	C4
Md1916	535	ACU13792.1: unknown [*Glycine max*]	1,00E-07	84	C1A
Md1800	97	AAO08997.1: unknown [*Vibrio vulnificus* CMCP6]	1,00E+00	88	unclustered
Md3502	350	CBI35038.1: unnamed protein product [*Vitis vinifera*]	1,00E-11	74	C1A
Md1792	141	CBI39978.1: unnamed protein product [*Vitis vinifera*]	1,00E-02	62	C3
