# Supplementary material for: Transcriptional analysis of cell growth and morphogenesis in the unicellular green alga Micrasterias (Streptophyta), with emphasis on the role of expansin
Source: BMC Plant Biol. 2011 Sep 25;11:128. doi: 10.1186/1471-2229-11-128 (PMC3191482; doi:10.1186/1471-2229-11-128)
Supplement: Additional file 5 — Comparison of the expression profiles of selected TDFs obtained by cDNA-AFLP and qRT-PCR for the samples of replication 2. [file 1471-2229-11-128-S5.PDF]

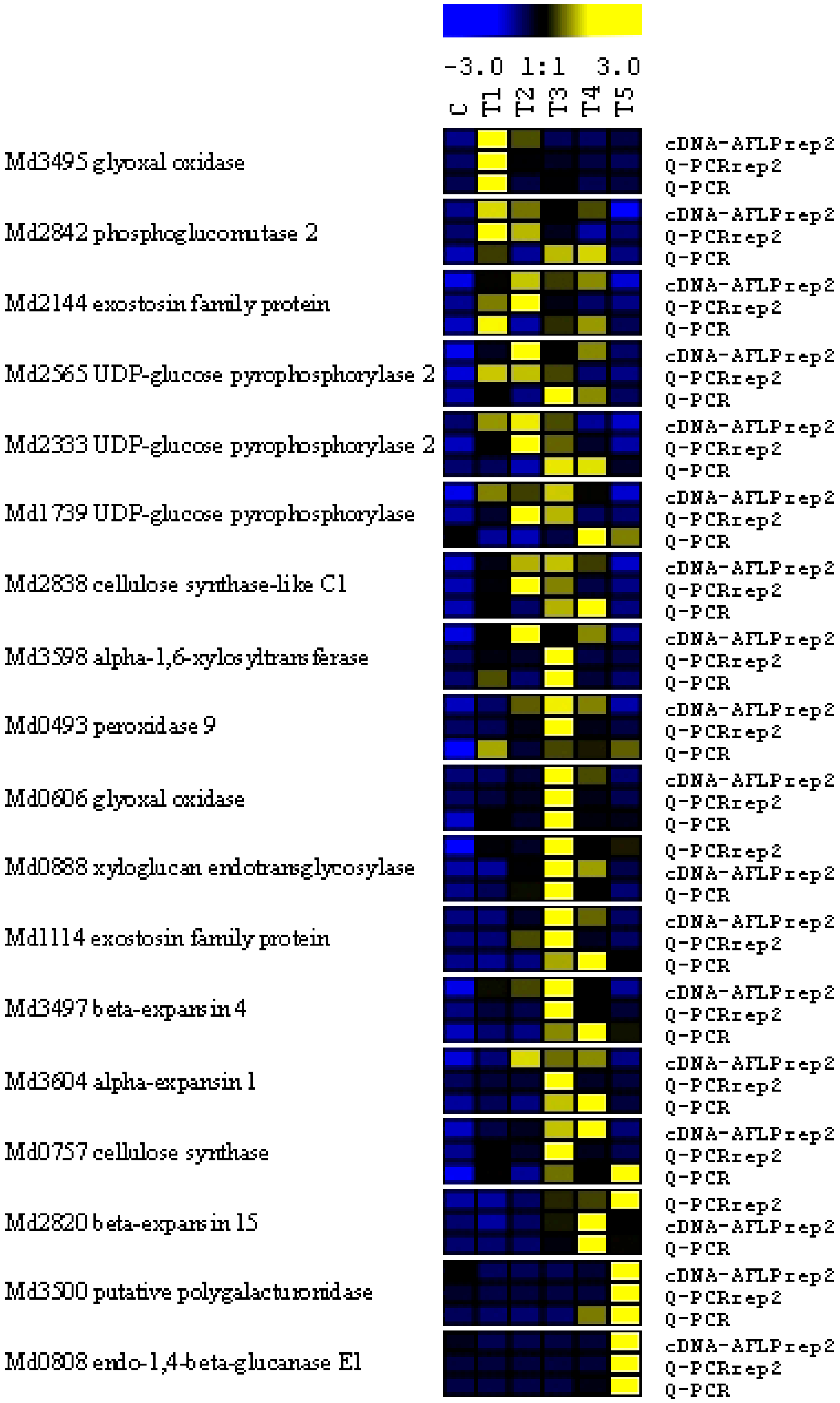

Additional file 5. Comparison of the expression profiles of selected TDFs obtained by cDNA-AFLP and qRT-PCR for the samples of replication 2. The qRT-PCR profile for each TDF of an independently sampled series is given in the third row.
