# Supplementary material for: Transcriptional analysis of cell growth and morphogenesis in the unicellular green alga Micrasterias (Streptophyta), with emphasis on the role of expansin
Source: BMC Plant Biol. 2011 Sep 25;11:128. doi: 10.1186/1471-2229-11-128 (PMC3191482; doi:10.1186/1471-2229-11-128)
Supplement: Additional file 7 — Full-length deduced amino acid sequence of MdXTH1 (Md0888) aligned with its relevant BLAST hits. [file 1471-2229-11-128-S7.PDF]

```
MdXTH1            M-----ASVMAALVLIAACAAPAMGVPRFRPRTAGATPGPTMACDTWTGAALCSVLPGGV 55
MaXET             M---------AMRFLLVACSLVAIASAG---------NFYQEFDVTWGGDRAKNLDNGQL 42
AtEXGT-A1         MTVSSSPWALMALFLMVSSTMVMAIPPRK----AIDVPFGRNYVPTWAFDHQKQFNGGSE 56
Pp_predicted      M---------DFVWMLGKYS-----------------KFQKSFFVAWSESNVAAVDGGHT 34
                  *             ::   :                         :*       .  *

MdXTH1            TELVLNKYGGGAMQIPNHYLTGQFSMDMKLPAGNSGGTATTFYLLDSITPTNKDPRHSEV 115
MaXET             LTLSLDKASGSGFQSKNQYLFGKIDMQIKLVPGNSAGTVTAYYLSS------QGPTHDEI 96
AtEXGT-A1         LQLILDKYTGTGFQSKGSYLFGHFSMHIKLPAGDTAGVVTAFYLSS------TNNEHDEI 110
Pp_predicted      LQLSLDRQSGTAVSSTSKYLYGYFRASIKLHSGNSAGTVTAFYLSS------QGHNHDEV 88
                    * *::  * ...  . ** * :   :** .*::.*..*::** .       .  *.*:

MdXTH1            DIEFFGNTTGNPMLFATNIFCGGFQN-LAQFRLPFDPAAAYHNYGFRWNTRQIVWLVDGQ 174
MaXET             DFEFLGNPSGDPYTLHTNVFTQGKGNREMQFKLWFDPTEDFHTYSILWNPRHVIFMVDGT 156
AtEXGT-A1         DFEFLGNRTGQPAILQTNVFTGGKGNREQRIYLWFDPSKAYHTYSILWNMYQIVFFVDNI 170
Pp_predicted      DFEFLGNVTGEPYVLQTNVYANGIGNREQRIFLWFDPRSEFHTYSVIWNHKSISMYVDDM 148
                  *:**:** :*:*  : **::  *  *   :: * ***   :*.*.. **   :   **.

MdXTH1            PIRVLNRTPRGPWPLLAMKPQ--------SSIWATPWTVIKPDFTFGPLKVFTKNFVAQG 226
MaXET             PIRDFKNLESRGIAFPNSQPMRIYSSLWNADDWATRGGLVKTDWSKAPFTASYRNFKADT 216
AtEXGT-A1         PIRTFKNAKDLGVRFPFNQPMKLYSSLWNADDWATRGGLEKTNWANAPFVASYKGFHIDG 230
Pp_predicted      LIRVFQNNEAHGQPYLSKQPMGVYSSIFDASNWATRGGLDKIDFNNAPFHAHYANFTMDS 208
                   ** ::.           :*         :. ***   : * ::  .*: .   .*  :

MdXTH1            CPALKTQVMAAAMVP-------RCAGAWNAQLTPVQLAAYARLRKATLVVDYGWDKA--- 276
MaXET             CVPSSATTECASNSVPS--NGGWWNQ----ELDSMGQQRMKWVQKNYMIYNYCSDLKRFS 270
AtEXGT-A1         CQASVEAKYCATQGR------MWWDQKEFRDLDAEQWRRLKWVRMKWTIYNYCTDRTRFP 284
Pp_predicted      CVVNETVTTSVADPCVAPTSTEWWNAEWFQSIPANRVGQMQWVNHNFMVYDYCTDKERFP 268
                  *        ..:                  .: .        :.    : :*  *

MdXTH1            -------------
MaXET             QGLPPECSIA--- 280
AtEXGT-A1         V-MPAECKRDRDA 296
Pp_predicted      V-APFECAAPIV- 279
```

**Additional file 7.** Full-length deduced amino acid sequence of *M. denticulata* MdXTH1 (GenBank accession number HE578718) aligned with protein sequences giving a significant BLAST hit: a xyloglucan endotransglycosylase from *Musa acuminata* [ABL10090.1], the endoxyloglucan transferase EXGT-A1 from *Arabidopsis thaliana* [NP_178708.1], and a predicted protein from *Physcomitrella patens* subsp. *patens* [XP_001775702.1]. Predicted secretion signal peptides are indicated in grey boxes in the alignment. The GH16-XET domain is indicated under the alignment with a black box, under the conserved catalytic site, the box is filled in grey. The possible N-linked glycosylation sites are boxed in the alignment. Tyrosines, putatively involved in acceptor substrate recognition are boldly underlined. Asterisks indicate identical residues; colons and periods indicate full conservation of strong and weak groups, respectively.
