# Supplementary material for: Transcriptional analysis of cell growth and morphogenesis in the unicellular green alga Micrasterias (Streptophyta), with emphasis on the role of expansin
Source: BMC Plant Biol. 2011 Sep 25;11:128. doi: 10.1186/1471-2229-11-128 (PMC3191482; doi:10.1186/1471-2229-11-128)
Supplement: Additional file 8 — Full-length deduced amino acid sequence of Md0434 aligned with its relevant BLAST hits. [file 1471-2229-11-128-S8.PDF]

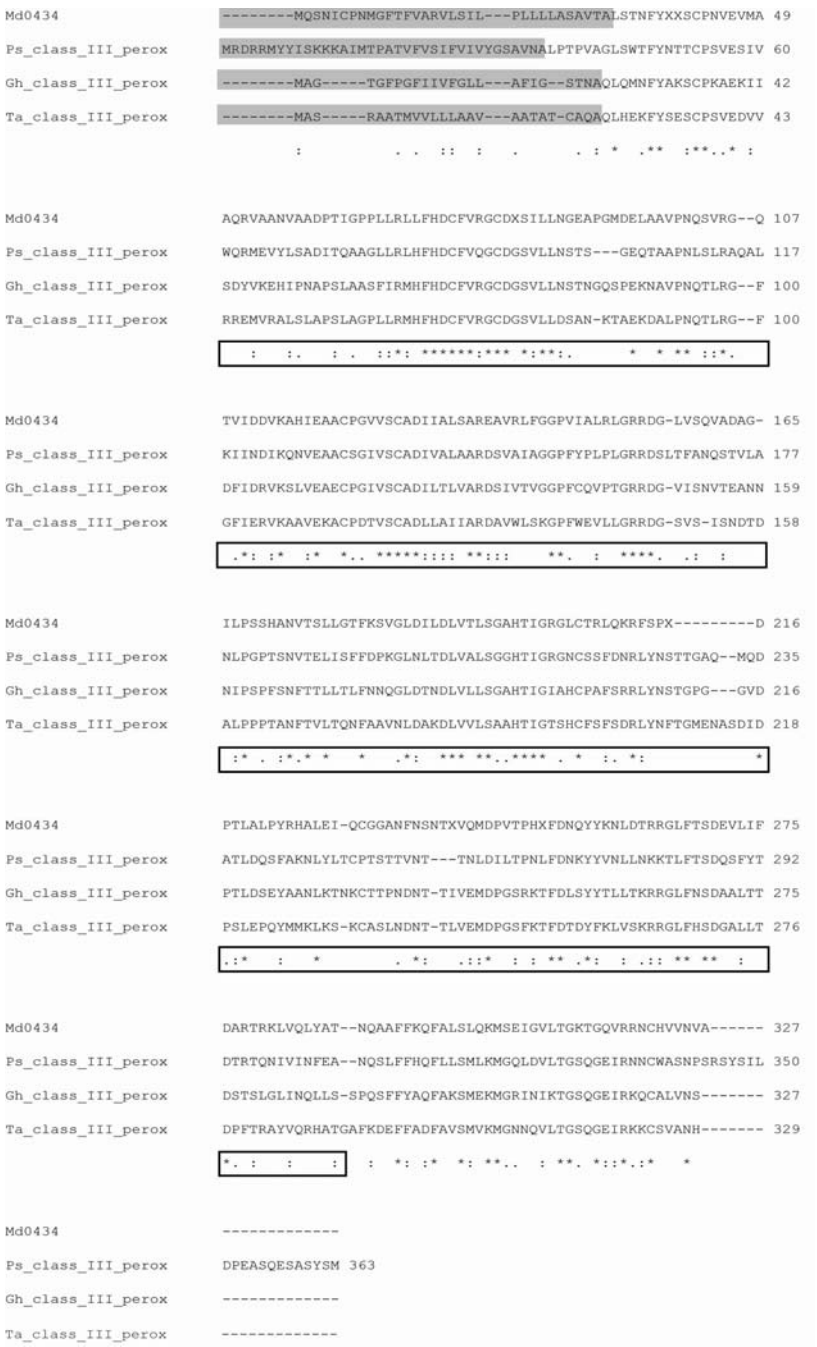


**Additional file 8.** Full-length deduced amino acid sequence of *M. denticulata* Md0434 (GenBank accession number HE578717) aligned with protein sequences giving a significant BLAST hit: a class III peroxidase from *Pinus sylvestris* [AAG02215.1], from *Gossypium hirsutum* [ACJ11761.1], and from *Triticum aestivum* [ACI00841.1]. Signal peptides are indicated in grey boxes in the alignment; the pfam peroxidase domain is indicated under the alignment with a black box. Asterisks indicate identical residues; colons and periods indicate full conservation of strong and weak groups, respectively.
