# Supplementary material for: Transcriptional analysis of cell growth and morphogenesis in the unicellular green alga Micrasterias (Streptophyta), with emphasis on the role of expansin
Source: BMC Plant Biol. 2011 Sep 25;11:128. doi: 10.1186/1471-2229-11-128 (PMC3191482; doi:10.1186/1471-2229-11-128)
Supplement: Additional file 9 — Characteristics of the expansin-resembling genes from Micrasterias denticulata. [file 1471-2229-11-128-S9.PDF]

Gene name	Accession number	Length (AA)	Molecular weight (kDa)	BLAST hit with annotated expansin	E-value	% similarity	% identity
MdEXP1 (Md1418)	HE578719	239	25.82	α-expansin EXPA1 [*Triticum aestivum*]	0.001	52	34
MdEXP2 (Md2820)	HE578720	448	46.47	*α-expansin precursor* [*Nicotiana tabacum*]	2.E-30	56	40
MdEXP3 (Md3497)	HE578721	296	30.93	α-expansin 5 (*Physcomitrella patens*)	3.E-25	63	39
MdEXP4 (Md3604)	HE578722	193	19.99	α-expansin 13 precursor, putative [*Ricinus communis*]	2.E-11	53	30

Additional file 9. Characteristics of the expansin-like genes from *Micrasterias denticulata*.
