# Supplementary material for: Transcriptional analysis of cell growth and morphogenesis in the unicellular green alga Micrasterias (Streptophyta), with emphasis on the role of expansin
Source: BMC Plant Biol. 2011 Sep 25;11:128. doi: 10.1186/1471-2229-11-128 (PMC3191482; doi:10.1186/1471-2229-11-128)
Supplement: Additional file 10 — Unrooted maximum likelihood phylogeny showing the relationship of putative chlorophytan expansin sequences (with significant similarity to plant expansins in tblastx searches) with the plant expansin gene family. [file 1471-2229-11-128-S10.PDF]

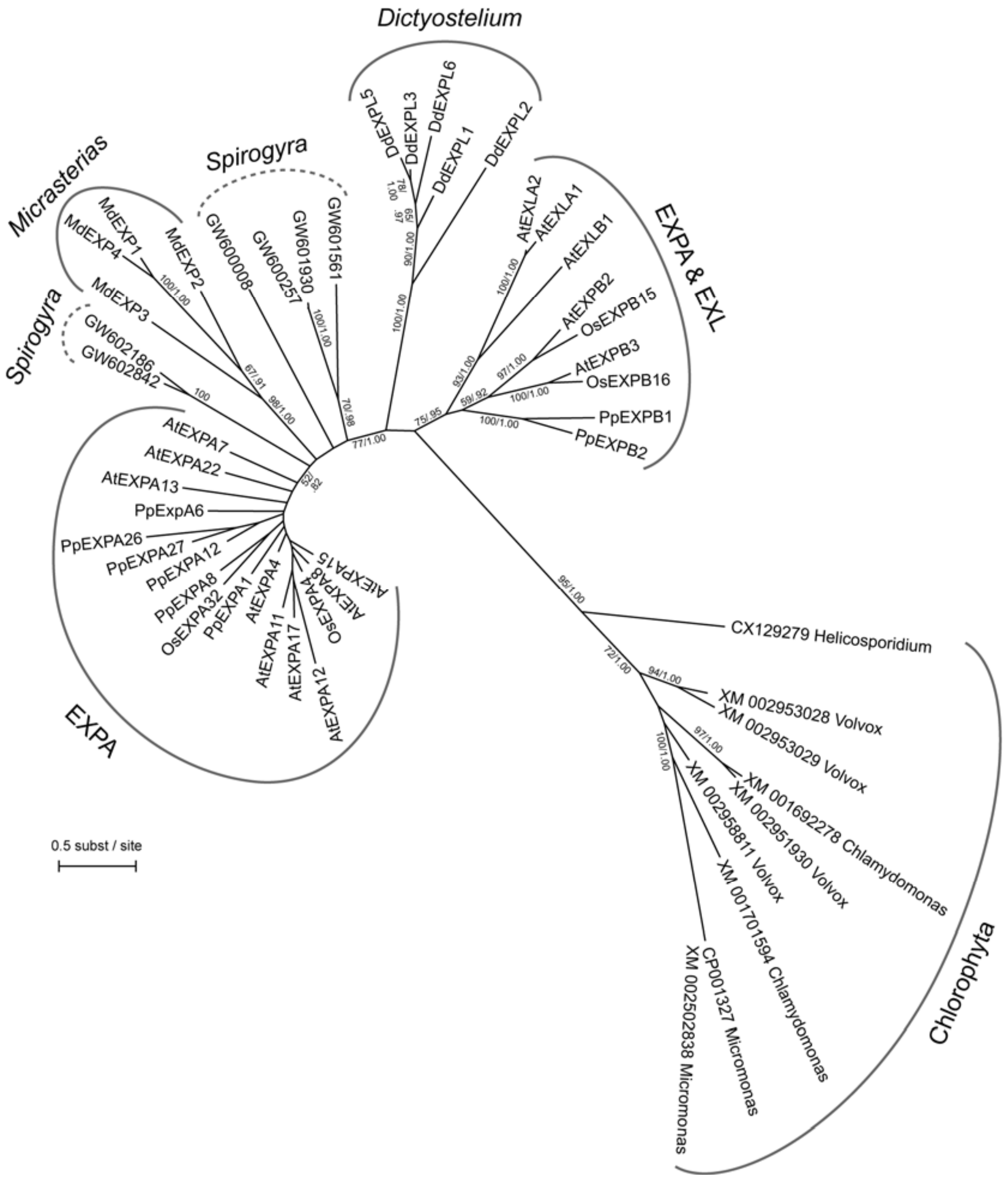

Additional file 10. Unrooted maximum likelihood phylogeny showing the relationship of putative chlorophytan expansin sequences (showing significant similarity to plant expansins in tblastx searches) with the plant expansin gene family. Numbers at nodes indicate ML bootstrap values and Bayesian posterior probabilities; values below respectively 50 and 0.9 are not shown.
