# Supplementary material for: Transcriptional analysis of cell growth and morphogenesis in the unicellular green alga Micrasterias (Streptophyta), with emphasis on the role of expansin
Source: BMC Plant Biol. 2011 Sep 25;11:128. doi: 10.1186/1471-2229-11-128 (PMC3191482; doi:10.1186/1471-2229-11-128)
Supplement: Additional file 11 — Protein BLAST alignment of MdEXP2 with its best hit, showing the expansin-like C-terminal extension. [file 1471-2229-11-128-S11.PDF]

```
ref|XP_002523388.1| Alpha-expansin 1 precursor, putative [Ricinus communis]
gb|EEF38967.1| Alpha-expansin 1 precursor, putative [Ricinus communis]
Length=247

GENE ID: 8282146 RCOM_0342250 | Alpha-expansin 1 precursor, putative
[Ricinus communis]

Score =  124 bits (311),  Expect = 2e-26, Method: Compositional matrix adjust
Identities = 81/222 (36%), Positives = 122/222 (54%), Gaps = 11/222 (4%)

Query  24   AIPTRDGLGTLSGVEKGGSCGFANNFP-APGVFTAGVSAAIYGNGAACGACFVATCANSP  82
            A  T  G G  SG   GG+CG+ N +    G  TA +S A++ +G +CGACF   C N
Sbjct  28   AHATFYGGGDASGT-MGGACGYGNLYSQGYGTNTAALSTALFNSGLSCGACFEIKCVNDN  86

Query  83   Q-CTANRVFFTVTNQC-----LGENSTSPCVTGRSGVALQPQAFDVIATSRAPGIVPVKF  136
            + C    +  T TN C     L  N+   C   +    L    F  IA  RA GIVPV++
Sbjct  87   KWCLPGSIIITATNFCPPNLALPNNNGGWCNPPQQHFDLSQPVFQRIAQYRA-GIVPVQY  145

Query  137  TQVPCRTAGGVQFVVQSGNQYYFAVLIQNVGGPGSLQAVAVSTNGRTFQLMTRSYGAVWQ  196
             +VPCR  GG++F + +G+ Y+  VLI NVGG G + AV++  +   +Q M+R++G  WQ
Sbjct  146  RRVPCRKTGGIRFTI-NGHSYFNLVLITNVGGAGDIVAVSIKGSNTNWQAMSRNWGQNWQ  204

Query  197  VSNFDIRRASLHFRLTGNDGQQLTILNALPANWVAKRIYSSL  238
             S++ +   SL F++T +DG+ +   NA P+NW   + Y+ +
Sbjct  205  SSSY-LNGQSLSFKVTTSDGRTVISNNAAPSNWAFGQTYTGM  245


Score = 70.5 bits (171),  Expect = 5e-10, Method: Compositional matrix adjust
Identities = 50/161 (31%), Positives = 82/161 (50%), Gaps = 11/161 (6%)

Query  287  GGACGFANYPS--VATLQAGLSETLYRNGAFCGSCLRVACVNSPQ-CIPGTVTVQVTNLC  343
            GGACG+ N  S    T  A LS  L+ +G  CG+C  + CVN  + C+PG++ +  TN C
Sbjct  43   GGACGYGNLYSQGYGTNTAALSTALFNSGLSCGACFEIKCVNDNKWCLPGSIIITATNFC  102

Query  344  TASNA--SAAMSVCVDGNPAVNLQPEAWDKIVKSRSPGVASVLFQQISCASPAQGVQFQV  401
              + A  +     C       +L    + +I + R+ G+  V ++++ C     G++F +
Sbjct  103  PPNLALPNNNGGWCNPPQQHFDLSQPVFQRIAQYRA-GIVPVQYRRVPCRKTG-GIRFTI  160

Query  402  RDANPTYFS-VLVQNVGGIGALTGVEVAFGGGGKWTAMARS  441
                 +YF+ VL+ NVGG G +  V +  G    W AM+R+
Sbjct  161  NGH--SYFNLVLITNVGGAGDIVAVSIK-GSNTNWQAMSRN  198
```

Additional file 11. Protein BLAST alignment of *Md*2820 (448 amino acids long) (GenBank accession number HE578720) with its best hit, showing that also its C-terminal extension is expansin-like.
