# Supplementary material for: Transcriptional analysis of cell growth and morphogenesis in the unicellular green alga Micrasterias (Streptophyta), with emphasis on the role of expansin
Source: BMC Plant Biol. 2011 Sep 25;11:128. doi: 10.1186/1471-2229-11-128 (PMC3191482; doi:10.1186/1471-2229-11-128)
Supplement: Additional file 12 — Confocal GFP fluorescence time lapse images (30 s apart) illustrating the motility of the MdEXP2-GFP containing intracellular compartments. [file 1471-2229-11-128-S12.PDF]

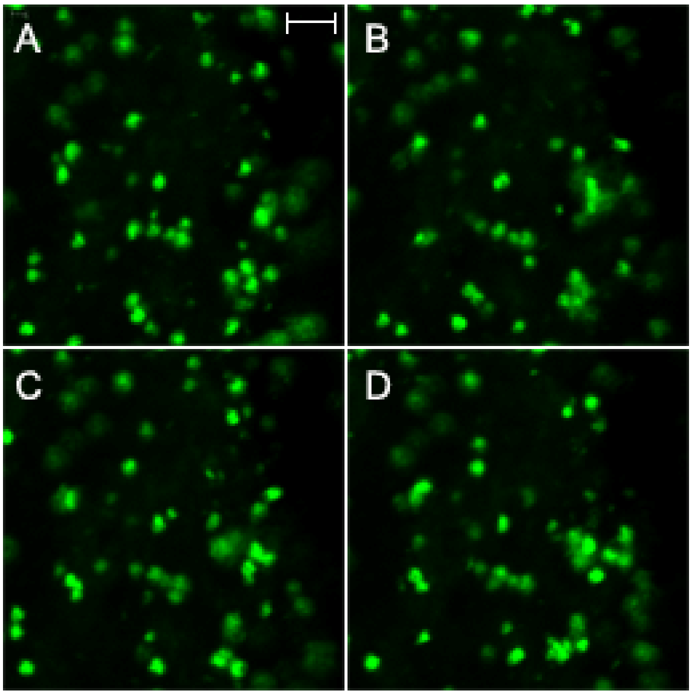

Additional file 12. Confocal GFP fluorescence time lapse images (30 s apart) illustrating the motility of the MdEXP2-GFP containing intracellular compartments. Scale bar = 5 µm.
