# Supplementary material for: Transcriptional analysis of cell growth and morphogenesis in the unicellular green alga Micrasterias (Streptophyta), with emphasis on the role of expansin
Source: BMC Plant Biol. 2011 Sep 25;11:128. doi: 10.1186/1471-2229-11-128 (PMC3191482; doi:10.1186/1471-2229-11-128)
Supplement: Additional file 13 — Features of transgenic cell lines overexpressing the MdEXP2-GFP fusion gene. [file 1471-2229-11-128-S13.PDF]

Additional file 13. Features of transgenic cell lines overexpressing the *MdEXP2-GFP* fusion gene. Cell lines are numbered according to their indicative GFP-fluorescence upon isolation, which is not a quantitative measurement. Cell fate: (D) no cell division, cell death; (L) loss of GFP-signal prior to cell division; or (P) cell division resulting in new semicells displaying a phenotype.

Cell line	Indicative GFP fluorescence	Fate	1st division phenotype	2nd division phenotype	Additional divisions displaying phenotype
1	37269	D			
2	70070	D			
3	73810	D			
4	76426	L			
5	76667	D			
6	78295	P	Fused lateral lobes, impaired axial elongation	(2x) Fused lateral lobes, impaired axial elongation (Fig. 8C)	No, loss of GFP signal
7	98590	P	Fused lateral lobes, impaired axial elongation	(1x) Fused lateral lobes, impaired axial elongation (Fig. 8D)	No, loss of GFP signal
8	107527	P	Fused lateral lobes, impaired axial elongation	(1x) Fused lateral lobes, impaired axial elongation	No, loss of GFP signal
9	121480	D			
10	138679	L			
11	150072	P	Elongated lobes (Fig. 8B)	(1x) Fused lateral lobes, impaired axial elongation	No, loss of GFP signal
12	154926	P	Fused lateral lobes, impaired axial elongation	(2x) Fused lateral lobes, impaired axial elongation	(2x) Fused lateral lobes, impaired axial elongation, finally loss of GFP signal
13	158453	P	Loss of growth planarity (Fig. 8F-H)	(1x) Loss of growth planarity, fused semicells (Fig. 8I)	No, cell death
14	170724	D			
15	171510	D			
16	197820	D			
17	204251	D			
18	240805	P	Fused lateral lobes, impaired axial elongation, fused new semicells (Fig. 8E)	No, cell death	
19	245499	P	Fused lateral lobes, impaired axial elongation	No, cell death	
20	253509	D			
21	417645	D			
22	431871	L			
23	467875	D			
24	521473	D			
25	536684	D			
26	635867	D			
