# Supplementary material for: Transcriptional analysis of cell growth and morphogenesis in the unicellular green alga Micrasterias (Streptophyta), with emphasis on the role of expansin
Source: BMC Plant Biol. 2011 Sep 25;11:128. doi: 10.1186/1471-2229-11-128 (PMC3191482; doi:10.1186/1471-2229-11-128)
Supplement: Additional file 14 — Primer sequences of selected Micrasterias denticulata TDFs used for real-time qRT-PCR assay. [file 1471-2229-11-128-S14.PDF]

Additional file 14. Primer sequences of selected *Micrasterias denticulata* TDFs used for real-time qRT-PCR assay.

TDF	sequence similarity	forward gene specific primer (5'-3')	reverse gene specific primer (5'-3')	product length (bp)
Md0493	class III peroxidase	CCAGCAGCACAGAGGCATC	ACAGGCAACGACACAAGGC	91
Md0606	glyoxal oxidase	GTCTGCGGGTATCATGTTTAGG	CAACCTGGTACTGGGTCTACG	123
Md0757	cellulose synthase	TGCTGCTGTCCTCTATCTTCTC	GCCATAAACTGAACCCACTCTG	128
Md0808	glycoside hydrolase	ATGGAGCCTCTATGGTGTAGTG	ACCGCCATCAGCAACAAGG	110
Md0888	xyloglucan endotransglycosylase	GAGTAAGGGGTCCAAAGGTG	CATGAAGCCCCAGTCCTC	77
Md1114	exostosin family protein	ATCCTTCCCCCATAAGTGAC	TATGGTGGAGGAGGTTTTGA	102
Md1739	udp-glucose pyrophosphorylase	GTCCTTCTTCCCCTTAGCAG	CGGAGCTGTGAGAATCTTGA	94
Md2144	exostosin family protein	ATGATCAACCATGGGCTTAG	GGAGTTGGCATCATTCAAAA	115
Md2333	udp-glucose pyrophosphorylase 2	ACCTCGAGACAACATGAAGG	TGAAAGTGGAGGCACCTAAG	148
Md2565	udp-glucose pyrophosphorylase 2	AAGGTGTCAGGAGATGTGGA	GCAAGTCAAAACAGCTCCAT	114
Md2820	β-expansin	CTCCGAATGCAACTTCAACT	ACGCCAACCCAACTTACTTT	84
Md2838	cellulose synthase-like protein c1	TTCCAGCAGCAACCAAGAGTAG	GCCAACCAGCCAGTGACAG	148
Md2842	cytosolic phosphoglucomutase	CCATCAGTCTTGGCAGTATGAG	TGGCACTGGGTCGGTAGG	75
Md3497	β-expansin	GCAAAGGTTGAGGTGTTGGTTC	ACCCAGAGGTAAGACGGAAGG	130
Md3495	glyoxal oxidase	GCCATTGACAACCACGAACAG	AACAATCACGGGATGAGAAACG	144
Md3500	putative polygalacturonidase	GTTGCCAGGCTTCCAGAGAG	GGGAACCGTAACCAACACCAG	75
Md3598	α-1,6-xylosyltransferase	AGTCCCTGAGTAAGAGGATGTG	GCGGGCGTTCAACTTTGC	144
Md3604	α-expansin	CCTGAACATTTCGTGGAAAC	ATGCAGGTAGTGGTCCAAAA	93
		**Normalization genes**		
Md0386	unknown gene at5g13390	GTACTCCGACTGTTTTGCGTAG	GTCCTCCTGTTGTGTGTTCCC	75
Md0789	reticulon	ACAGCCAACAGCAATCAAAGC	TATGTGAATGGGAAGGCGGATG	101
Md1473	peroxiredoxin	TCTCCCTCCTTCCAGTCCAC	AACGAGATTCTGCGGGTGATAG	83
