# Supplementary material for: Transcriptional analysis of cell growth and morphogenesis in the unicellular green alga Micrasterias (Streptophyta), with emphasis on the role of expansin
Source: BMC Plant Biol. 2011 Sep 25;11:128. doi: 10.1186/1471-2229-11-128 (PMC3191482; doi:10.1186/1471-2229-11-128)
Supplement: Additional file 15 — Primers used for RACE PCR and cloning. [file 1471-2229-11-128-S15.PDF]

Additional file 15. Primer sequences used for RACE PCR.

TDF	Forward gene specific primer (5'-3')	Reverse gene specific primer (5'-3')	Reaction
*Md*4341	GCGTCTCTTCTACTGGTTCTGG	CAATGCCAAGGATTACCACTTT	First RACE PCR
	TATCAGTGGCACATCGTACACA	ACCAGTCAGGATCTTTGCATTT	Second RACE PCR
	TCAGGAACCAAAGTGGTAATCC	TGTGTACGATGTGCCACTGATA	Sequencing
*Md*XTH1	GTTCTACCTCCTCGACAGCATC	GTCAGGTTTGATTACGGTCCAG	First RACE PCR
	GGAAGTGGACATCGAGTTTTTC	GAAAAACTCGATGTCCACTTCC	Second RACE PCR
	AAACCTGACTTCACCTTTGGAC	CAGCTTCATATCCATCGAGAAC	Sequencing
MdEXP2	TGCAACTTCAACTCCAGTAAGC	ATTTTGCACCAGTACGGAAAAG	First RACE PCR
	ATTTTGCACCAGTACGGAAAAG	AGCGCTTACTGGAGTTGAAGTT	Sequencing
	GAGCGCTTACTGGAGTTGAAGT	GAGAACGGCGAAGTAGTACTGG	Sequencing
MdEXP1		CCCTCCTAAACCATCAACAGAG	First RACE PCR
		CCTGTACATCGGGAAAAACTTC	Second RACE PCR
		ATGAGAACCTGCATGTAAGCAA	Sequencing
MdEXP4	AGTCGTGTTCAACGTCTCAGTG	GATGGTGAGAATTCTGTTGTGC	First RACE PCR
	TACATGCAGGTAGTGGTCCAAA	TCCTGAACATTTCGTGGAAAC	Second RACE PCR
	GCACAACAGAATTCTCACCATC	GCGTACACTTCAATAGGCACAG	Sequencing
MdEXP3		GGAGATCAAGAGGGTAGCATTG	First RACE PCR
		TACATGCCTGAGATAGCATTGG	Second RACE PCR
		AAAAGTTCACGTTTGCCAGATT	Sequencing
*Md*0434		GGAGGTCCAATTGAAGACTTTG	First RACE PCR
		CTCCGTACTTGACCAGTTTTCC	Second RACE PCR
		GTCAAAAATGAGCACCTCATCA	Sequencing
pDONR222.1	ACGACGGCCAGTCTTAAGCTCGG	ACGACGGCCAGTCTTAAGCTCGG	Sequencing
