# Supplementary material for: Transcriptional analysis of cell growth and morphogenesis in the unicellular green alga Micrasterias (Streptophyta), with emphasis on the role of expansin
Source: BMC Plant Biol. 2011 Sep 25;11:128. doi: 10.1186/1471-2229-11-128 (PMC3191482; doi:10.1186/1471-2229-11-128)
Supplement: Additional file 16 — Accession numbers of the sequences used to construct the phylogenetic trees and additional Physcomitrella patens sequences. [file 1471-2229-11-128-S16.PDF]

Additional file 16. Accession numbers of the sequences used to construct the phylogenetic trees and additional *Physcomitrella patens* sequences

ORGANISM	PROTEIN NAME	ACCESSION NUMBER
Dictyostelium discoideum	Expansin-like protein 1	Q55G31.1
Dictyostelium discoideum	Expansin-like protein 2	Q54PA4.1
Dictyostelium discoideum	Expansin-like protein 3	Q7KWS2.1
Dictyostelium discoideum	Expansin-like protein 5	Q86AV4.1
Dictyostelium discoideum	Expansin-like protein 6	Q55G32.1
Dictyostelium discoideum	Expansin-like protein 7	Q54J35.1
Physcomitrella patens	Expansin A6	AAN08122.1
Arabidopsis thaliana	Expansin A4	NP_181500.1
Arabidopsis thaliana	Expansin A7	NP_172717.1
Arabidopsis thaliana	Expansin A8	NP_181593.1
Arabidopsis thaliana	Expansin A11	NP_173446.1
Arabidopsis thaliana	Expansin A12	NP_188156.1
Arabidopsis thaliana	Expansin A13	NP_566197.1
Arabidopsis thaliana	Expansin A15	NP_178409.2
Arabidopsis thaliana	Expansin A17	NP_192072.1
Arabidopsis thaliana	Expansin A22	NP_198743.2
Arabidopsis thaliana	Expansin B2	NP_564860.3
Arabidopsis thaliana	Expansin B3	NP_001078454.1
Arabidopsis thaliana	Expansin-like A1	NP_190183.1
Arabidopsis thaliana	Expansin-like A2	NP_195553.1
Arabidopsis thaliana	Expansin-like B2	NP_193436.2
Oryza sativa	Expansin A4	AAL24481.1
Oryza sativa	Expansin A32	Q6YYW5.1
Oryza sativa	Expansin B15	AAM73779.1
Oryza sativa	Expansin B16	Q0DZ85.1

Physcomitrella patens Expansin A1

MARHNATKPVTLILAALMVLSATDNVEGRHHVRDGKNWRKAHATFYGGADASGTMGGACGYGNLYSTGYGVDSTALSTALF
NNGAKCGACFAIQCYRSQYCVPGSPVITVTATNFCPPNHKGDGTPGCNPPMRHFDLAQPSFTKIAKYRAGIVPVLFRRVPCEKKG
GVRFTINGNKYFNLVLVHNVGGKGDVHAVDIKGSNTEWIPMKRNWGMNWQTDAVMTGQALSFRVTTSDGKTIVSMNATPSH
WSFGQTFGGQFAMN

Physcomitrella patens Expansin A1

MAKFSAQIVVAFMVLLAQQVRAESGWNEAHATFYGGSDAGGTTGTRGGACGYGDLYSTGYGTSTVAISSALFDRGLACGACYQ
VKCAGSSSECRSDSPAIQVTVTNFCPPNPSLPEDNGGWCNLPLHFDMSMPAFEQIATYKAGIVPVMYRRTSCVRTGGIHFTMSGH
NFMNLVLVTNVGGMGDVQSVSIRGSKTSWVTMTRNFGQIWQSTVNMSGQSLSFMVTTSDGKTVVSNNVAPPDWAFGQTYEGS
QF

Physcomitrella patens Expansin A1

MALRARQSSVLEVVVLVIMFGCVRMEGVEAWSGPNGWNDAHATYYGGADASGGACGFGNLYSTGYGTSTAALSQALFNSGLT
CGACFELACDPSGSKYCYKGSSIVVTATNFCPSGSEGGWCDSPKQFDLSQPVFNKIAQQAGGVIPVKYRRVPCRKSGGMRFTING
NPYFILVLVTNVGGAGDVQQLSLKGSSTGWYTMSRNWGQQWEFRGNSALVGQALSFRVVTSDGAEAVSYDAASENWSFSQTFE
GINF

Physcomitrella patens Expansin A1

MKMSATSGVQLAVLLLALSVSSVLGGPFGWNFGRITYYGSPNGGGTQGGACGYQNTFALGYGTNTAALSSRLFQGGAACGACY
QLRCIAPKWGKNWCWNYARSIVVTATNLCPSGSNGGWCPSQAHF?PMPAFTSLARKEGGVTPIMYRRVRCARRGGIRFTIGGNPF
FLMVLIHNVGGAGDVRAVSIKGQYTGWVGMYRNWGSLWTCTTKIDGALTFRITTGDGKTLILYNAVRKGWRFGQTWEGSQFR

Physcomitrella patens Expansin A1

MTIEQRTMVQAHVTFKLLLVLAGCVLQVQAAYGPSGWATATATFYGGADAGGGACGYGNLYSTGYGASTTALSAPL?NGGSA
CGACYQLQCARSNHCYAGRSITVTATNFCPTGSEGGWCNPPRKHFDLSMPMFTTLARQVAGVVPVDYRRVACNKKGGQRFLMT
GNPYFIMVLVYNVAGAGDVQRFFVKGSMTGWYELRRNWGIWTCTADSRLKGQALSFRTQTSDGRQVVSIDAAPANWNFGQTF
SSGVN

Physcomitrella patens Expansin A1

MGGFQLGLLLLCCVVGVSQAWDLPGGVKLSAEGYNENWLGGHATWYGDPYGEGSSGGACGYTQLTGTPIGSKIAAGNAPIFQE
GKGCGQCYEVKCNYPSCSPQGTRIVITDLCPGGQYCSTDQPAFDSGAAITAMALPGRDGELRNIGLYDIQYKRVPCEYPNQNIAFK
VDAGSSKYWFSFTVKYLGGPGDINTVEVKCGKNGYFQYAQHSWGANWMLINYSGVPFQFPLTIKITTKLNDHTVVAEDVIPDWF
GGVQYESNVQIRY

Physcomitrella patens Expansin A1

MSTAFQAVWLVCVGLLSLQAAEAGYLAQNGYHERWVRARGTWYGDPYGEGSSGGNCGYTKLWGTPIGPKIVAGSRSIYANGQ
GCGQCYQIRCVDPNGGPRLCNPQGTNVVVTDFCPGGTYCSTGENADMSGAAINAMALRGRE
