# Supplementary material for: Transcriptional analysis of cell growth and morphogenesis in the unicellular green alga Micrasterias (Streptophyta), with emphasis on the role of expansin
Source: BMC Plant Biol. 2011 Sep 25;11:128. doi: 10.1186/1471-2229-11-128 (PMC3191482; doi:10.1186/1471-2229-11-128)
Supplement: Additional file 6 — Full-length deduced amino acid sequence of Md4341 aligned with its relevant BLAST hits. [file 1471-2229-11-128-S6.PDF]

```
Md4341   --MGSFQLASLLLVLALSLLSSEARISRPATTT---------------LAKDLDAAFAKL 43
BsFLA    -MELKGAIFTTAVLATLLCSPALAQKSPPAPASVALPPTMAPAPAPAPDYVDLAALLGVA 59
AtAGP    MATSRTFIFSNLFIFFLVLATTYGQAPAPGPS----------------GPTNITAILEKA 44
                : :  .:  *   .: .: . *..:                   :: * :

Md4341   AKTKGYS-IISGTSYTSQVKKAVLASGVKTITFLAPNDAASQAAEQN--PDLISAGGTTV 100
BsFLA    GPFHTFLSYLQKTNVIETFQRQAN-KTKEGITIFVPKDSAFAALKKSTFSNLTSDQLKTL 118
AtAGP    GQFTLFIRLLKSTQASDQINTQLNSSSSNGLTVFAPTDNAFNSLKSGTLNSLSDQQKVQL 104
         .    :   :. *.  . .:     .  : :*.:.*.* *  : :..   .* .     :

Md4341   VEYHTFPTLYTATQLSGLPANAKILTGAG----SSMIKTSAQGKTPVKLKSPKAKAVATV 156
BsFLA    LLYHAFPEFYSLAQFRNLSVLNPVNTFAG----APYTLNLTDDMGTISVKS--MWSKPTI 172
AtAGP    VQFHVLPTLITMPQFQ--TVSNPLRTQAGDGQNGKFPLNITSSGNQVNITT--GVVSATV 160
         : :*.:* : : .*:   ..   : * **    .    . :..   :.:.:      .*:

Md4341   IQTVYSGTKVVILGIDTVLLPA-------------------------------------- 178
BsFLA    SSSVYATDPVAVYSLNKVLLPMQIFTKDPPLAPAPAPAPESGASDLAPSPGGKAGAGKGK 232
AtAGP    ANSVYSDKQLAVYQVDQVLLPLAMFG--SSVAPAPAPEKGGSVSKGSASGGDDGGDSTDS 218
          .:**:   :.: ::  ****

Md4341   -----------------------------
BsFLA    ADSTSAAYGIGVGAVNCLLAAGGLMMLLW 261
AtAGP    SDAERTAFGFGIRITTVAAIAASSSLWI- 246
```

Additional file 6. Full-length deduced amino acid sequence of *Micrasterias denticulata Md*4341 (GenBank accession number HE578723) aligned with protein sequences giving a significant BLAST hit: a fasciclin-like protein from *Brachypodium sylvaticum* [CAJ26371.1] and an arabinogalactan protein-like from *Arabidopsis thaliana* [AAM62616.1]. Signal peptides are indicated in grey boxes in the alignment; the fasciclin domain is indicated under the alignment with a black box. Asterisks indicate identical residues; colons and periods indicate full conservation of strong and weak groups, respectively.
